# Bevacizumab and anlotinib combination therapy acts via HIF-1α suppression to exert synergistic anti-angiogenic and anti-tumor effects in non-small cell lung cancer

**DOI:** 10.3389/fimmu.2025.1613368

**Published:** 2025-09-17

**Authors:** Nafeisha Simayi, Jiaying Li, Junkai Hu, Ning Jia, Rongxue Wan, Jinbiao Xie, Wenhua Huang

**Affiliations:** ^1^ Guangdong Engineering Research Center for Translation of Medical 3D Printing Application, Guangdong Provincial Key Laboratory of Digital Medicine and Biomechanics, National Key Discipline of Human Anatomy, School of Basic Medical Sciences, Southern Medical University, Guangzhou, China; ^2^ Orthopaedic Center, Affiliated Hospital of Guangdong Medical University, Guangdong Medical University, Zhanjiang, China; ^3^ School of Medicine, Yan ‘an University, Yan ‘an, China; ^4^ Department of Cardiothoracic Surgery, Affiliated Hospital of Putian University, Putian, China

**Keywords:** microfluidic chip, non-small cell lung cancer, bevacizumab, anlotinib, angiogenesis, epithelial–mesenchymal transition

## Abstract

**Introduction:**

Tumor angiogenesis is required for the progression of non-small cell lung cancer (NSCLC), and anti-vascular endothelial growth factor (anti-VEGF) antibody bevacizumab and multitarget tyrosine kinase inhibitor anlotinib are anti-cancer treatment options, the combined effect of which in NSCLC remains unclear.

**Methods:**

A vascularized microfluidic chip was applied to model angiogenesis, together with *in vitro* assays, molecular analyses, and an *in vivo* mouse xenograft model, to evaluate therapeutic effects. Immunological changes were examined by assessing T-cell infiltration and cytokine levels, and the role of HIF-1α was validated using an inhibitor and an activator.

**Results:**

Bevacizumab plus anlotinib (B+A) inhibited angiogenesis, reducing vessel density to 10% of control values and also reducing diameter and green fluorescent protein (GFP) area ratio. B+A inhibited cell viability by 78%, colony formation by 90%, and invasion by 75% in NSCLC cell lines A549 and H1299; downregulated N-cadherin 5.34-fold, vimentin 6.46-fold, and α-SMA 4.35-fold; and upregulated E-cadherin 3.75-fold. The rates of apoptosis of A549 and H1299 cells were increased 3.85-fold. The phosphorylation of VEGFR2, PDGFRβ, and FGFR1 was also reduced. B+A reduced tumor volume 7.23-fold and weight 7.08-fold, decreased tumor cell density, and lowered Ki-67 expression in an *in vivo* mouse xenograft model. HIF-1α was inhibited in a time- and dose-dependent manner. Importantly, the combination therapy enhanced CD4^+^ and CD8^+^ T-cell infiltration, increased the production of pro-inflammatory cytokines such as IL-2, and reduced the expression of immunosuppressive factors such as IL-6, indicating an immunomodulatory effect that improved anti-tumor immunity.

**Conclusion:**

HIF-1α inhibitor PX478 did not enhance the anti-tumor effects of B+A, but HIF-1α activator DMOG reversed them. In addition, the combination therapy enhanced CD4^+^ and CD8^+^ T-cell infiltration and increased pro-inflammatory cytokines. These findings highlight the therapeutic potential of combining anlotinib and bevacizumab for NSCLC treatment and identify HIF-1α as a key target.

## 1 Introduction

Non-small cell lung cancer (NSCLC) accounts for approximately 85% of all lung cancer cases, and there are over 2 million new diagnoses per annum (1–3). The 5-year survival rate for advanced NSCLC patients remains below 20% despite advances in surgery, chemotherapy, radiotherapy, immunotherapy, and targeted therapies, illustrating the need for more therapeutic options (4–6). Tumor heterogeneity due to genetic variation both within and between primary and metastatic sites complicates treatment and contributes to the development of drug resistance (7). NSCLC growth and metastasis depend on the generation of new blood vessels by angiogenesis (8), making this process an anti-cancer target exploited by drugs, such as bevacizumab (9, 10), a monoclonal antibody that inhibits vascular endothelial growth factor (VEGF) (11). Bevacizumab extended progression-free survival in advanced NSCLC patients (12), but its therapeutic efficacy is often limited by the activation of compensatory proangiogenic pathways, leading to resistance and reduced long-term effectiveness (13, 14). Anlotinib is an anti-angiogenic multitarget tyrosine kinase inhibitor (TKI) that inhibits receptor signaling for VEGF, fibroblast growth factor (FGF), and platelet-derived growth factor (PDGF) (15). The multitarget nature of anlotinib raises the possibility of its enhancing the anti-tumor effect of bevacizumab and overcoming resistance mechanisms (16–18). However, the molecular mechanisms by which these two therapeutic agents may interact and the impact on signaling pathways, such as the PI3K/AKT/HIF-1α axis, remain unclear (19, 20).

The HIF-1α transcription factor mediates cellular responses to various stressors, including hypoxia, which characterizes many tumors *in vivo* (21, 22), and stimulates angiogenesis, metabolic reprogramming, and the epithelial–mesenchymal transition (EMT) (23–26). All these processes are considered to impact tumor growth and progression, and PI3K/AKT signaling represents an upstream regulator of HIF-1α, promoting its stability and activation under hypoxia (27, 28). HIF-1α mediates the transcription of proangiogenic factors, enhancing tumor vascularization and metastasis (29). Any involvement of PI3K/AKT/HIF-1α signaling in the synergistic anti-tumor effects of anlotinib and bevacizumab may expose new therapeutic targets and biomarkers.

*In vitro* models that mimic the complex vascular environment have been enabled by microfluidic technology and present a great advantage over two-dimensional cell culture systems in the study of tumor angiogenesis and the assessment of anti-angiogenic treatments (30–32). Angiogenesis, nutrient diffusion, and drug responses may be recapitulated in a dynamic manner within a controlled environment (33–36). In the present study, a novel vascularized microfluidic model is presented and validated by testing the antiangiogenic actions of anlotinib and bevacizumab. The effect of HIF-1α in mediating the synergistic anti-tumor effects of these drugs was investigated, and anti-tumor actions were evaluated *in vitro* in A549 and H1299 NSCLC cell lines and *in vivo* in a mouse xenograft model. The aim was to illustrate the contribution of microfluidic modeling to the study of angiogenesis and to indicate the benefits of combined therapy with bevacizumab and anlotinib (B+A) for NSCLC treatment, highlighting the potential for novel therapeutic targets resulting from increased molecular understanding.

## 2 Materials and methods

### 2.1 Microfluidic device fabrication

Microfluidic devices were fabricated with a three-layered architecture comprising a glass substrate, polydimethylsiloxane (PDMS) structural layer, and a polymethyl methacrylate (PMMA) reservoir layer. Computer-aided design (CAD) software was used to design one central gel channel (8 µL) and two adjacent medium channels with inlet diameters of 1 and 1.5 mm, which were patterned onto a silicon wafer master mold via soft lithography and photolithography. PDMS prepolymer (Sylgard 184, Dow Corning, Midland, MI, USA) was mixed with a curing agent at a 10:1 ratio, degassed to eliminate air bubbles, poured over the master mold, and cured at 60 °C for approximately 2 h. The PDMS layer was peeled off the mold, bonded to a clean glass substrate, and pretreated with oxygen plasma for 30 seconds to increase surface hydrophilicity and promote strong adhesion. The PDMS–glass assembly was plasma-treated for 30 seconds to ensure a robust and leak-proof seal. The PMMA reservoir layer was fabricated by laser cutting (Epilog Laser, Golden, CO, USA) with 40 µL of reservoir volume for the central gel channel and 240 µL for the medium channels with 1- and 1.5-mm inlet diameters. The PMMA layer was integrated by adhesive bonding (Loctite 406, Henkel, Düsseldorf, Germany) onto the PDMS–glass assembly with the alignment of inlet holes and microchannels. The complete microfluidic device was inspected under a microscope (Olympus IX73, Olympus Corporation, Tokyo, Japan) to verify channel dimensions and bond integrity, and leak tests were performed by introducing dye solution (Sigma-Aldrich, St. Louis, MO, USA) into the inlet reservoirs.

### 2.2 Cells and reagents

Primary human umbilical vein endothelial cells expressing GFP (GFP-HUVECs) and primary normal human lung fibroblasts (NHLFs) were purchased from Lonza (Basel, Switzerland). GFP-HUVECs were cultured in endothelial growth medium-2 (EGM-2; Lonza) and NHLFs in fibroblast growth medium-2 (FGM-2; Lonza). NSCLC cell lines A549 (human lung adenocarcinoma) and H1299 (human large cell lung carcinoma) were purchased from American Type Culture Collection (ATCC; Manassas, VA, USA) and cultured in Roswell Park Memorial Institute medium (RPMI-1640, Gibco, Waltham, MA, USA) supplemented with 10% fetal bovine serum (FBS; Gibco) and 1% penicillin–streptomycin (Gibco). Routine cell culture was performed at 37 °C in a humidified incubator with 5% CO_2_ except for dose-dependent experiments examining the proteins of the PI3K/AKT/HIF-1α pathway, HIF-1α time-dependent experiments, and experiments involving PX478 and DMOG, which were performed in the presence of 1% CO_2_. Cell lines were profiled by short tandem repeat (STR) sequencing and confirmed to be mycoplasma-free.

A 5-µM stock solution of HIF-1α inhibitor PX-478 (Selleck Chemicals, Houston, TX, USA) and a 200-µM stock solution of HIF-1α activator dimethyloxalylglycine (DMOG; Sigma-Aldrich) were prepared in dimethyl sulfoxide (DMSO). Anlotinib (Selleck Chemicals) and bevacizumab (Selleck Chemicals) were also dissolved in DMSO. All stock solutions were stored at −20 °C and diluted with culture media to ensure a final DMSO concentration ≤ 0.1%. A DMSO vehicle control was included in all experiments.

### 2.3 Cell culture in microfluidic device

GFP-HUVECs and NHLFs at passages 3–5 were cultured to over 80% confluence, detached with trypsin–ethylene diamine tetraacetic acid (EDTA) (0.05% trypsin, 0.53 mM EDTA; Gibco), and resuspended in culture media; 7 × 10^5^ GFP-HUVECs and 2 × 10^5^ NHLFs were centrifuged at 300 × *g* for 5 minutes at room temperature, the supernatant was discarded, and the cell pellet was resuspended in 100 μL Vascular Chip Perfusion Solution I (Danwang Medical, Shanghai, China). Vascular Perfusion Solution II (Danwang Medical) at a volume of 2 μL was mixed with 8 μL cell suspension, ensuring the absence of bubbles, and slowly introduced into the central gel channel inlet port, allowing surface tension to immobilize the cells within the central gel channel. The microfluidic device was incubated at 37 °C with 5% CO_2_ for 10 minutes to facilitate cell adhesion, 300 μL endothelial growth medium-2 was added to one side channel, and 200 μL fibroblast growth medium-2 was added to the opposite side channel. Culture media were changed every 2 days. Approximately 6 days after seeding, altered culture media levels were seen in the side channels, and the device was placed on a programmable Bluetooth shaker (Model I24101, Danwang Medical) oriented with flow channels perpendicular to the direction of oscillation with a 2-h time interval and a 10° angle. The culture medium was changed every 2 days.

### 2.4 Drug administration in microfluidic chips

Anti-angiogenic agents, 10 μM anlotinib and 200 μg/mL bevacizumab, were administered 10 days after seeding either individually or in combination with DMSO as a control. Culture media containing drugs were continuously perfused through the medium channels to maintain consistent drug exposure and mimic physiological perfusion conditions. Chips were incubated for 4 days, during which vascular perfusion assays, permeability assays, and structural quantification were performed.

### 2.5 Quantification of blood vessel density, area, diameter, and node count

The fluorescence microscopy images of GFP-HUVECs were processed using the ImageJ software to perform background subtraction, thresholding, and skeletonization and to estimate blood vessel area as the GFP area ratio (GFAR). Total vascular length and sprout diameter were assessed, and nodes were counted using the particle analysis function of the Analyze Skeleton plugin. Data were averaged across multiple random fields of view, and statistical analysis was performed using one-way analysis of variance (ANOVA) with Tukey’s post-hoc test in GraphPad Prism. A value of *p* < 0.05 was considered statistically significant.

### 2.6 Vascular perfusion and permeability assays

#### 2.6.1 Vascular perfusion

Ten days after cell seeding, the medium was removed, 300 µL 70-kDa dextran conjugated with red fluorescent protein (RFP) (Danwang Medical) in phosphate-buffered saline (PBS) was introduced via the side channel/reservoir, and fluorescence was monitored under a microscope for 10 seconds.

#### 2.6.2 Permeability

DAPI-labeled fluorescent microspheres (Danwang Medical) at a volume of 100 µL were diluted with 900 µL PBS, the medium was removed from the chip, fluorescent microsphere solution was added via the side channel, and real-time fluorescence microscopy was used to visualize and quantify microsphere movement through the vascular network for 10 seconds. Fluorescence images were quantified using the ImageJ software, and statistical analysis was performed using one-way ANOVA and a significance threshold of *p* < 0.05.

### 2.7 C_50_ determination

Cell Counting Kit-8 (CCK-8; Dojindo Laboratories, Kumamoto, Japan) assays were used to determine half-maximal inhibitory concentrations (IC_50_) of anlotinib and bevacizumab in A549 and H1299 cell lines, with analysis using the GraphPad Prism 9.0 software. The percentage of viable cells was plotted against log drug concentration, and IC_50_ values were calculated by fitting data to a non-linear regression sigmoidal dose–response curve with a variable slope. All IC_50_ determinations were performed in triplicate.

### 2.8 Cell viability assay

Cell viability was assessed by CCK-8 assay according to the manufacturer’s protocol. A549 and H1299 cells were seeded into 96-well plates at a density of 5 × 10^3^ cells per well and allowed to adhere overnight before the addition of 600 μg/mL bevacizumab and/or 10 μM anlotinib for viability measurements at 0, 24, 48, and 72 h. Briefly, 10 µL CCK-8 reagent was added per well, the plates were incubated for 2 h at 37 °C, and absorbance was measured at 450 nm using a microplate reader (BioTek Instruments, Winooski, VT, USA). All experiments were performed in triplicate, and data were analyzed using the GraphPad Prism software. Viability is expressed as a percentage of the untreated control group. Statistical significance was determined using one-way ANOVA followed by Tukey’s post-hoc test with a threshold of *p* < 0.05.

### 2.9 Colony formation assay

A549 and H1299 cells were seeded into six-well plates at a density of 1,000 cells per well, allowed to adhere overnight before the addition of bevacizumab and/or anlotinib, and cultured for 14 days with medium replacement every 3 days. Colonies were fixed with 4% paraformaldehyde for 15 minutes, stained with 0.1% crystal violet for 20 minutes, and counted under a light microscope. Colony formation is expressed as the number of colonies formed as a percentage of the number of cells seeded. All experiments were conducted in triplicate, and statistical analysis was performed using one-way ANOVA with Tukey’s post-hoc test with a threshold of *p* < 0.05.

### 2.10 Apoptosis assay

Apoptosis was assessed using the Annexin V-FITC/PI Apoptosis Detection Kit (BD Biosciences, San Jose, CA, USA) following the manufacturer’s protocol. A549 and H1299 cells were seeded into six-well plates at a density of 1 × 10^5^ cells per well and allowed to adhere overnight before the addition of bevacizumab and/or anlotinib for 24 h. Cells were harvested, washed twice with cold PBS, resuspended in 1× binding buffer, and incubated with annexin V-FITC and propidium iodide (PI) for 15 minutes at room temperature in the dark. Apoptosis was quantified by flow cytometry (BD FACSCanto II, BD Biosciences), and data were analyzed using the FlowJo software to determine percentages of early (annexin V^+^/PI^−^) and late (annexin V^+^/PI^+^) apoptotic cells. All experiments were performed in triplicate with statistical analysis using one-way ANOVA at a threshold of *p* < 0.05.

### 2.11 Cell invasion assay

A549 or H1299 cells and HUVECs were used for invasion assays. For tumor cells, 1 × 10^5^ A549 or H1299 cells in 250 μL serum-free medium were seeded into the upper chamber of an 8 μm pore size Transwell insert (Corning, NY, USA) coated with Matrigel (Corning) in the presence of bevacizumab (600 μg/mL), anlotinib (10 μM), or their combination. The drugs were present in the upper chamber with the cell suspension, while the lower chamber contained 500 μL complete medium with 10% FBS. Chambers were incubated at 37 °C and 5% CO_2_ for 48 h. Non-invasive cells on the upper membrane surface were removed using a cotton swab, and invasive cells on the lower membrane surface were fixed with 4% paraformaldehyde for 15 minutes and stained with 0.1% crystal violet for 20 minutes. For HUVECs, cells were pretreated with bevacizumab (600 μg/mL), anlotinib (10 μM), or their combination for 48 h prior to the invasion assay. Subsequently, 2 × 10^4^ treated HUVECs suspended in serum-free medium were seeded into the upper chamber of Matrigel-coated Transwell inserts (8 μm pore size), with the lower chamber containing medium supplemented with 10% FBS as a chemoattractant. After incubation at 37 °C for 24 h, non-invading cells were removed from the upper membrane surface, and invaded cells were fixed and stained as above. For both cell types, invaded cells were counted in five randomly selected fields under a light microscope. All experiments were conducted in triplicate with statistical significance determined using one-way ANOVA at a threshold of *p* < 0.05.

### 2.12 Migration assay

HUVECs were cultured to 90%–95% confluence in six-well plates. A sterile 200-μL pipette tip was used to create a uniform scratch (wound) across the cell monolayer. The wells were gently washed twice with PBS to remove detached cells and replaced with serum-free medium containing the indicated treatments. The images of the scratch area were captured immediately (0 h) and at 24 h after treatment with different groups using an inverted microscope. The migration rate was quantified by measuring the wound closure area using the ImageJ software and is expressed as a percentage of the initial scratch area. Experiments were performed in triplicate, and statistical significance was assessed using one-way ANOVA, with *p* < 0.05 considered significant.

### 2.13 Enzyme-linked immunosorbent assay

A549 and H1299 cells (2.0 × 10^5^ cells/well) were seeded into six-well plates and, after overnight attachment, treated with the indicated conditions for 48 h, with the final 24 h in low-serum medium (0.5%–1% FBS). Culture supernatants were collected, centrifuged at 300 × *g* for 5 min, and stored at −80 °C until analysis. The levels of TGF-β, TNF-α, IFN-γ, IL-2, IL-10, IL-8, and IL-6 were measured using human enzyme-linked immunosorbent assay (ELISA) kits (R&D Systems, Minneapolis, MN, USA) according to the manufacturer’s instructions. Absorbance was read at 450 nm with a 570-nm reference using a microplate reader, and concentrations were calculated from standard curves and normalized to total protein content. All assays were performed in triplicate, and statistical analysis was conducted using one-way ANOVA with *p* < 0.05 considered significant.

### 2.14 Quantitative real-time polymerase chain reaction

Total RNA was extracted from A549 and H1299 cells using the TRIzol reagent (Invitrogen, Carlsbad, CA, USA) according to the manufacturer’s protocol, contaminated genomic DNA was removed by DNase digestion, RNA was quantified using the NanoDrop spectrophotometer (Thermo Fisher Scientific, Waltham, MA, USA), and RNA with an A260/A280 ratio between 1.8 and 2.0 was used. cDNA was synthesized from 1 µg total RNA by reverse transcription using the PrimeScript RT Reagent Kit (Takara, Shiga, Japan) following the manufacturer’s instructions. Quantitative real-time polymerase chain reaction (qRT–PCR) was performed using the SYBR Green PCR Master Mix (Applied Biosystems, Foster City, CA, USA) on an ABI 7500 Real-Time PCR System (Applied Biosystems) with the following reaction conditions: initial denaturation at 95 °C for 10 minutes, followed by 40 cycles of 95 °C for 15 seconds and 60 °C for 1 minute. The relative expression of genes encoding E-cadherin, N-cadherin, vimentin, α-SMA, HIF-1α, Ang2, GLUT1, LDHA, and PDK1 was normalized to GAPDH expression using the 2^−ΔΔCt^ method. Primer sequences (**Supplementary Table S1**) were designed using Primer-BLAST and synthesized by Sangon Biotech (Shanghai, China). All experiments were performed in triplicate, and data were analyzed using the GraphPad Prism software. Statistical significance was determined using one-way ANOVA with Tukey’s post-hoc test at a threshold of *p* < 0.05.

### 2.15 Western blotting

Total protein was extracted from A549 and H1299 cells with Radio Immunoprecipitation Assay (RIPA) lysis buffer (Beyotime, Shanghai, China) containing protease and phosphatase inhibitors (Roche, Basel, Switzerland), and protein concentration was determined using the BCA protein assay kit (Thermo Fisher Scientific). Equal amounts of protein (20–30 µg) were separated by sodium dodecyl sulfate-polyacrylamide gel electrohoresis (SDS–PAGE) on 10% or 12% polyacrylamide gels and transferred onto polyvinylidene fluoride (PVDF) membranes (Millipore, Burlington, MA, USA). Membranes were blocked with 5% skimmed milk in Tris-buffered saline with 0.1% Tween-20 (TBS-T) for 1 h at room temperature, incubated with primary antibodies overnight at 4 °C (**Supplementary Table S2**), washed three times with TBS-T for 10 minutes each, and incubated with horseradish peroxidase (HRP)-conjugated secondary antibodies (1:5,000, Cell Signaling Technology ,Danvers, MA, USA) for 1 h at room temperature. Protein bands were visualized using an enhanced chemiluminescence (ECL) reagent (Thermo Fisher Scientific) by ChemiDoc Imaging System (Bio-Rad, Hercules, CA, USA). Band intensities were quantified using the ImageJ software and normalized to β-actin or GAPDH. All experiments were performed in triplicate, and statistical analysis was conducted using one-way ANOVA with a threshold of *p* < 0.05.

### 2.16 *In vivo* experiments

A human NSCLC tumor xenograft model was established in female immunodeficient BALB/c nude mice (5–6 weeks old, 18–22 g) by subcutaneous injection of 2 × 10^5^ A549 cells into the right flank. Based on the high tumorigenicity of A549 cells in immunodeficient mice, a xenograft model was established using this cell line. When the tumor reached a volume of approximately 150 mm^3^, mice were randomly divided into four groups (n = 4): control (0.1% DMSO in PBS), bevacizumab (5 mg/kg bevacizumab given by intraperitoneal injection, twice weekly), anlotinib (1 mg/kg anlotinib given by oral gavage, daily), and B+A combination therapy (5 mg/kg bevacizumab + 1 mg/kg anlotinib administered as above). Tumor size was measured using a digital caliper every 3 days, animals were weighed weekly and sacrificed after 28 days, and tumors were harvested for hematoxylin and eosin (H&E) staining to assess morphology and for immunohistochemical (IHC) staining (n = 4, total 16). Western blotting analysis was conducted on fresh tissue. All animal experiments were conducted in accordance with institutional guidelines and approved by the Animal Ethics Committee (approval no.: SYT2024084). It can be confirmed that the maximal tumor size in this study did not exceed the tumor burden permitted by the Animal Ethics Committee.

### 2.17 TUNEL immunofluorescence staining

Paraffin-embedded tumor tissue sections (4 μm thick) from four treatment groups (n = 4 per group) were deparaffinized in xylene and rehydrated from graded ethanol to distilled water. After antigen retrieval in citrate buffer (pH 6.0) by microwave heating for 15 minutes, sections were washed with PBS and permeabilized with 0.3% Triton X-100 in PBS for 15 minutes at room temperature. Apoptotic cells were detected using the *In Situ* Cell Death Detection Kit, TMR red (Roche, Basel, Switzerland), according to the manufacturer’s instructions. Following terminal deoxynucleotidyl transferase-mediated dUTP-biotin nick end labeling (TUNEL) labeling, sections were blocked with 5% bovine serum albumin (BSA) in PBS for 30 minutes at room temperature and then incubated overnight at 4 °C with primary antibodies against endothelial marker CD31 (1:100, Abcam, Cambridge, UK), epithelial marker CK7 (1:200, Cell Signaling Technology, Danvers, MA, USA), or stromal marker α-SMA (1:400, Sigma-Aldrich). After washing, sections were incubated with appropriate Alexa Fluor-conjugated secondary antibodies (Invitrogen) for 1 h at room temperature in the dark. Nuclei were counterstained with DAPI (Thermo Fisher). Fluorescence images were captured using a confocal laser scanning microscope. The quantification of TUNEL-positive cells co-localizing with CD31, CK7, or α-SMA was performed in five randomly selected fields per section using the ImageJ software.

### 2.18 H&E staining

Tumor samples were fixed in 10% neutral-buffered formalin, embedded in paraffin, and sectioned into 4-µm slices. Sections were deparaffinized in xylene, rehydrated with ethanol gradient, rinsed with distilled water, mounted on resin-coated slides, stained with hematoxylin for 5 minutes, washed with running tap water, and counterstained with eosin for 2 minutes. Stained sections were observed under a light microscope (Leica Microsystems, Wetzlar, Germany) at ×200 magnification to assess tumor morphology and structure.

### 2.19 IHC staining

Paraffin-embedded tumor sections were deparaffinized and rehydrated as described previously. Antigen retrieval was performed in citrate buffer (pH 6.0) by heating in a microwave for 15 minutes. Sections were cooled to room temperature, rinsed with PBS, incubated with 3% hydrogen peroxide for 10 minutes to inhibit endogenous peroxidase activity, and blocked with 5% BSA in PBS for 30 minutes at room temperature. Sections were then incubated overnight at 4 °C with primary antibodies against Ki-67 (1:200, CST), CD4 (1:200, Abcam, Cambridge, UK), CD8 (1:200, Abcam), p-VEGFR2 (1:100, CST), p-PDGFRβ (1:100, CST), or p-FGFR1 (1:100, CST). After washing with PBS, sections were incubated with HRP-conjugated secondary antibody (1:500, CST) for 1 h at room temperature. Immunoreactivity was visualized using 3,3′-diaminobenzidine (DAB) substrate solution (Abcam) followed by counterstaining with hematoxylin. Sections were then dehydrated and mounted with coverslips. The quantification of Ki-67 expression and immune cell infiltration (CD4^+^ and CD8^+^ T cells) was performed by calculating the percentage of positively stained cells or positive cell counts per five randomly selected high-power fields per section using the ImageJ software. Phosphorylated receptor levels were assessed semi-quantitatively by evaluating staining intensity and positive cell percentage in the same fields.

### 2.20 Statistical analysis

All data are expressed as mean ± standard deviations (SDs) for at least three independent experiments. Statistical analyses were performed using the GraphPad Prism 9.0 software (GraphPad Software, La Jolla, CA, USA), and intergroup differences were analyzed using one-way ANOVA followed by Tukey’s post-hoc test for multiple comparisons. Comparisons between two groups were made using an unpaired two-tailed Student’s t-test, with a value of *p* < 0.05 considered statistically significant. Graphs were generated using GraphPad Prism.

## 3 Results

### 3.1 Development and functional validation of the vascularized microfluidic chip platform

**Figure 1:**
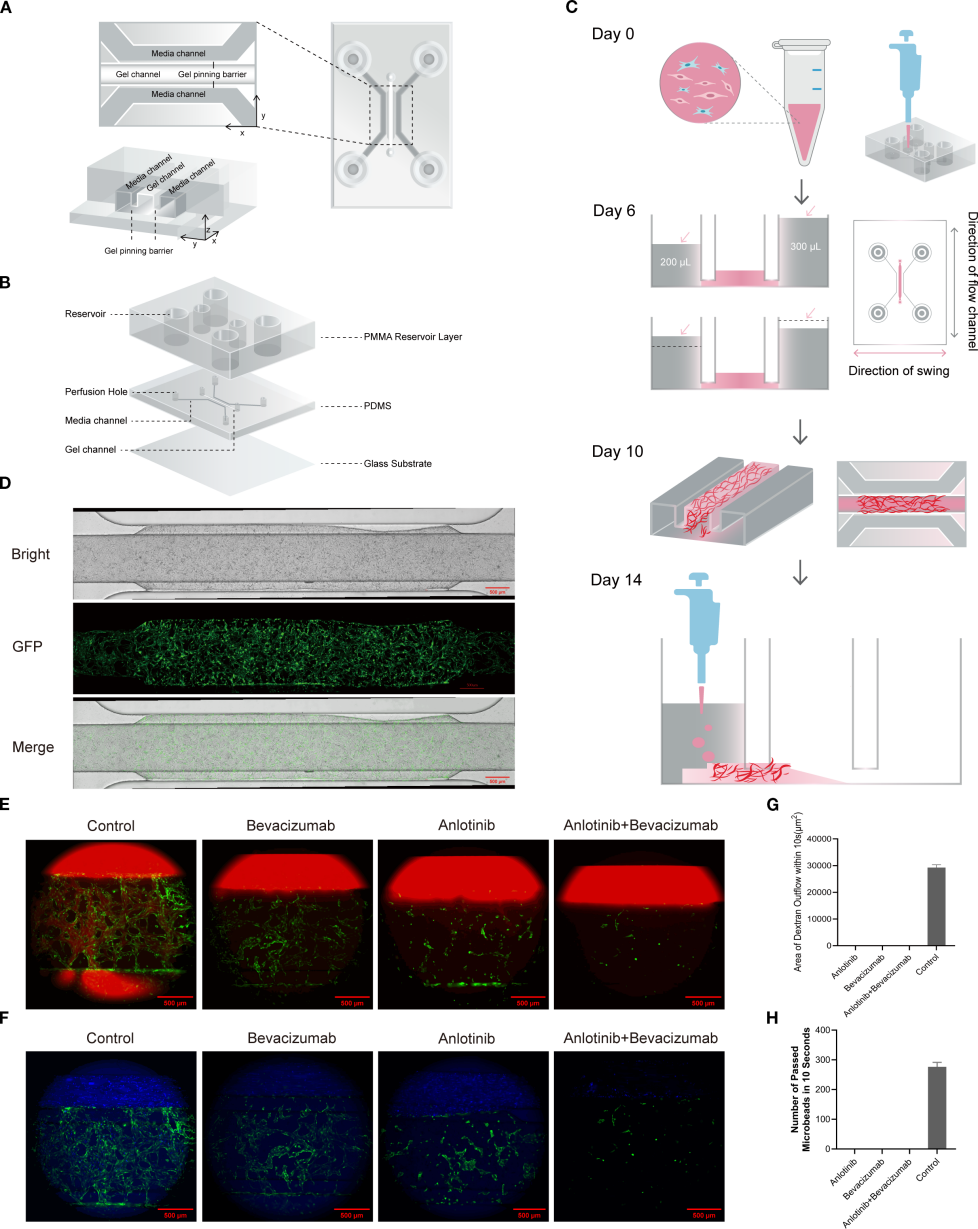
Structure and functional validation of the vascularized microfluidic chip. **(A)** Schematic representation of chip construction showing gel channel, media channels, and gel pinning barriers. The cross-sectional view highlights the alignment of the channels in the X, Y, and Z planes. **(B)** Three-dimensional structural overview of the chip, including PMMA reservoirs, PDMS channels, and glass substrate. **(C)** Timeline for vascular formation under experimental conditions: Day 0, GFP-HUVEC and NHLF seeding; Day 6, media level changes observed, with chip placed on programmable shaker (medium flow direction perpendicular to oscillation); Day 10, dense vascular network observed; Day 14, functional validation. **(D)** Brightfield and GFP fluorescence images of the vascular network on Day 10 demonstrating a continuous, interconnected structure. **(E)** Dextran perfusion assay results showing diffusion within the vascular network for control, bevacizumab, anlotinib, and B+A combination treatment. **(F)** Quantitative analysis of dextran diffusion area demonstrating reduced permeability in drug-treated groups compared to the control. **(G)** Fluorescent microbead perfusion assay in control, bevacizumab, anlotinib, and B+A combination treatment. The control group demonstrated continuous flow, but no flow was observed after combination treatment. **(H)** Quantitative analysis of microbead passage over 10 seconds showed significant disruption of vascular functionality in drug-treated groups. PMMA, polymethyl methacrylate; PDMS, polydimethylsiloxane; GFP-HUVEC, human umbilical vein endothelial cell expressing GFP; NHLF, normal human lung fibroblast.

In this study, microarray models were developed in order to investigate the angiogenic process and for the screening of anti-vascular drugs. The chip had a central gel channel flanked by two media channels separated by gel pinning barriers, ensuring stable gel injection and cell culture conditions to recapitulate the microvascular environment. The multilayer structure of PDMS layers, PMMA reservoirs, and a glass substrate created a dynamic microenvironment with continuous perfusion to facilitate vascular development (**Figures 1A, B**). A 3D schematic view and structural overview are shown in **Supplementary Figure S1**. GFP-HUVECs and NHLFs were co-seeded into the gel channel, and active endothelial sprouting and fibroblast-mediated gel contraction were indicated by differences in media levels between the media channels within 6 days. Oscillations perpendicular to the medium flow direction promoted vascular maturation. A dense, interconnected vascular network was established after 10 days of culture, at which point 10 μM anlotinib and 200 μg/mL bevacizumab were administered. Functional assays performed 4 days later confirmed vascular integrity and functionality (**Figure 1C**). Brightfield and fluorescence imaging showed a continuous, well-organized vascular network with successful angiogenesis shown by GFP-labeled endothelial cells (**Figure 1D**).

Fluorescent dextran perfusion of control chips seeded with untreated cells demonstrated the presence of the intact and permeable structures expected for functional vasculature. By contrast, treatment with anlotinib and/or bevacizumab abolished dextran diffusion, indicating disrupted vascular permeability and loss of functional vasculature (**Figures 1E, F**; Movies S1–S4). Fluorescent microbead perfusion showed the passage of approximately 286 microbeads through the control, untreated chip in 10 seconds compared with no microbead passage in any drug-treated group (**Figures 1G, H**; Movies S5–S8). Thus, a vascularized microfluidic chip that enabled angiogenesis and the production of a functional vascular network was successfully generated.

### 3.2 Evaluation of the anti-angiogenic effects of tumor drugs

**Figure 2:**
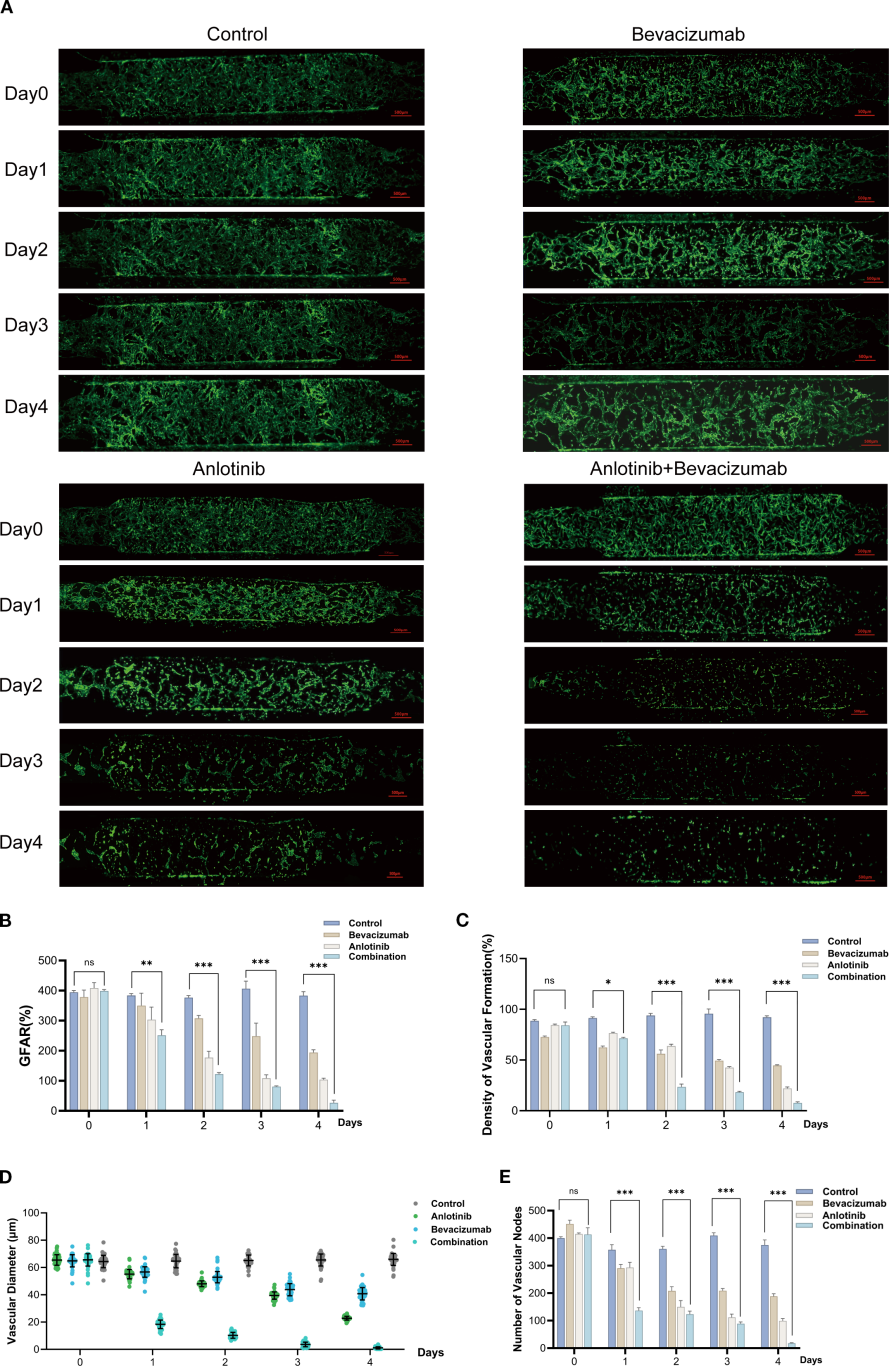
Anti-angiogenic effects of anlotinib and bevacizumab assessed using vascularized microfluidic chip. **(A)** Representative GFP images showing temporal changes over 4 days in vascular networks in control, anlotinib, bevacizumab, and combination B+A treatment groups. **(B)** Temporal changes in GFP area ratio (GFAR) for all treatment groups. **(C)** Vascular density as a percentage of control for anlotinib, bevacizumab, and B+A combination treatment. **(D)** Vascular diameter for all treatment groups. **(E)** Vascular node number over time for all treatment groups. Data are presented as mean ± SD for three independent experiments. *p < 0.05, **p < 0.01, and ***p < 0.001.

Vascular responses were monitored via assessment of GFAR, vascular density, vascular diameter, and vascular node count over a 4-day period of treatment with anlotinib and bevacizumab. The vascular network remained dense and continuous in untreated control conditions, but treatment with anlotinib or bevacizumab caused progressive fragmentation and reduced vascular density, which was apparent by Day 3. The impact was more pronounced for combined anlotinib and bevacizumab therapy, with significant disruption of vascular structures by Day 2 and near-complete loss of vascular integrity by Day 4 (**Figure 2A**). GFAR and vascular density at Day 4 declined to 40%–50% of control values with individual anlotinib or bevacizumab treatment, and a steep drop to less than 10% was seen for combined treatment. Vascular diameter decreased from a control value of approximately 60 μm to 30–35 μm for single-drug treatments and to 10 μm for combined therapy. The number of vascular nodes decreased from a control value of approximately 500 nodes to fewer than 50 with combined treatment (**Figures 2B–E** and **Supplementary Figures S2A–D**). Anlotinib and bevacizumab demonstrated a synergistic impact in disrupting vascular integrity and functionality, which could be quantitatively assessed in the microfluidic chip model, indicating the potential for this system in evaluating the efficacy of anti-tumor drugs (37).

### 3.3 Anti-tumor effects of anlotinib plus bevacizumab in NSCLC

**Figure 3:**
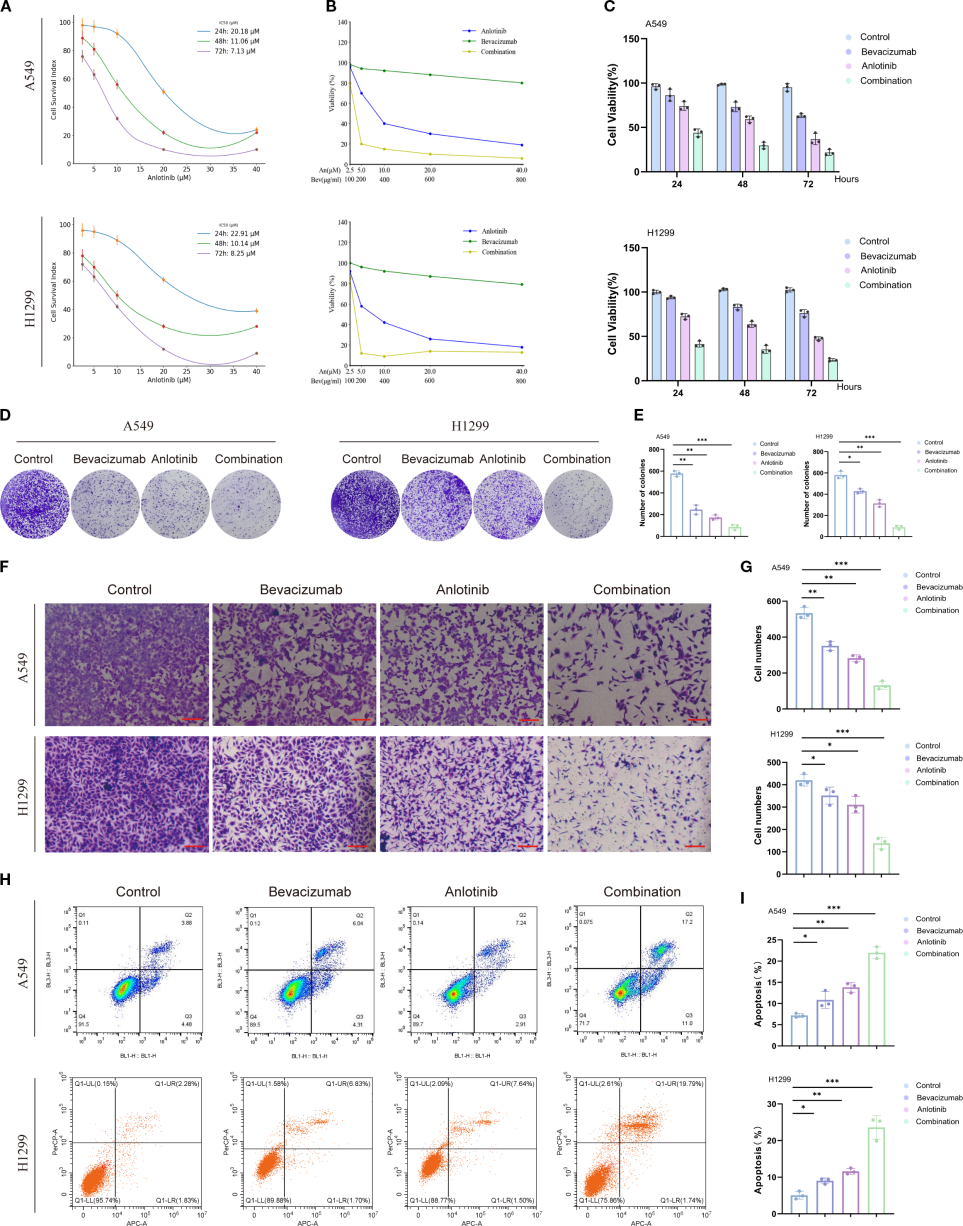
Effects of anlotinib, bevacizumab, and B+A combination treatment on survival, colony formation, and apoptosis of A549 and H1299 cells. **(A)** Drug sensitivity analysis showing a time-dependent decrease in anlotinib IC_50_ values for A549 and H1299 cells. **(B)** Dose-dependent effects of anlotinib and bevacizumab on viability of A549 and H1299 cells. **(C)** Viability of A549 and H1299 cells after treatment with 10 μM anlotinib, 600 μg/mL bevacizumab, or a combination of 10 μM anlotinib and 600 μg/mL bevacizumab for 24, 48, and 72 h. **(D)** Colony formation assays for A549 and H1299 cells with 10 μM anlotinib, 600 μg/mL bevacizumab, or a combination of 10 μM anlotinib and 600 μg/mL bevacizumab. **(E)** Quantitative analysis of colony formation in A549 and H1299 cells. **(F)** Representative images of Transwell invasion assays 48 h after treatment with 10 μM anlotinib, 600 μg/mL bevacizumab, or a combination of 10 μM anlotinib and 600 μg/mL bevacizumab. **(G)** Quantitative analysis of Transwell invasion assays for A549 and H1299 cells. **(H)** Rates of apoptosis of A549 and H1299 cells after 24-h treatment with 10 μM anlotinib, 600 μg/mL bevacizumab, or a combination of 10 μM anlotinib and 600 μg/mL bevacizumab. **(I)** Quantitative analysis of apoptosis in A549 and H1299 cells. Scale bar = 250 μm. Data are presented as mean ± SD for three independent experiments. *p < 0.05, **p < 0.01, and ***p < 0.001.

Drug sensitivity analyses of A549 and H1299 cells revealed a time-dependent decrease in IC_50_ values for anlotinib, with values decreasing from 20.18 μM at 24 h to 7.13 μM at 72 h in A549 cells and from 22.91 μM at 24 h to 8.25 μM at 72 h in H1299 cells, indicating increased sensitivity over time (**Figure 3A**). Both anlotinib and bevacizumab led to decreased cell viability after 24 h, rising to a 34%–58% reduction after 72 h, with the combination of the two drugs showing a stronger inhibitory effect with a 78% reduction after 72 h (**Figures 3B, C**). Single-drug treatments also reduced colony formation, with a reduction almost to zero for the combination therapy (**Figures 3D, E**). Invasive cell behavior was reduced by individual treatment with anlotinib (by 26%–47% for the two cell types) and bevacizumab (by 16%–34%), with the most potent effect seen for combined therapy (by 67%–75%; **Figures 3F, G**). Measurements of apoptosis showed that anlotinib increased the numbers of apoptotic cells by 91%–128% relative to controls and bevacizumab by 50%–77%, and the greatest effect was seen for the combination of the two drugs at 205%–365% (**Figures 3H, I**). The data show that anlotinib and bevacizumab have a synergistic effect in reducing proliferation, invasion, and colony formation and in inducing apoptosis in A549 and H1299 cells. Previous studies have demonstrated individual anti-tumor effects for anlotinib and bevacizumab (38, 39), but the synergistic effect makes the combination of the two drugs more potent.

The invasive capacity of HUVECs was evaluated using the Transwell invasion assay. In the control group, the number of invaded cells was the highest, averaging ~370 cells per field. Bevacizumab and anlotinib monotherapies reduced invasion to ~260 and ~240 cells, respectively, representing decreases of ~30%–35% compared with the control. The bevacizumab and anlotinib combination treatment produced a marked further reduction, with only ~120 invaded cells observed, which was significantly lower than either monotherapy. The addition of HIF-1α inhibitor PX478 to the combination group did not result in a statistically significant change compared with the combination alone, indicating that maximal suppression had been reached. In contrast, the addition of HIF-1α activator DMOG partially reversed the inhibitory effect, increasing the number of invaded cells to ~200 (**Supplementary Figure S3**). These results suggest that the anti-invasive effect of bevacizumab plus anlotinib on endothelial cells is, at least in part, mediated by the suppression of HIF-1α signaling.

The effect of the treatments on HUVEC migration was evaluated using a wound healing assay. After 24 h, the control group exhibited the highest migratory capacity with wound closure of approximately 69%. Bevacizumab and anlotinib monotherapies moderately reduced migration to ~58% and ~48%, respectively, corresponding to reductions of ~11% and ~21%, compared with the control. The bevacizumab and anlotinib combination treatment markedly suppressed migration, resulting in wound closure of only ~17%, which was significantly lower than either monotherapy. The Combination + PX478 group showed no significant difference compared with the combination group, whereas the Combination + DMOG group partially reversed the inhibitory effect, increasing wound closure to ~39% (**Supplementary Figure S4**). These findings indicate that bevacizumab plus anlotinib synergistically inhibits HUVEC migration and that this effect can be partially attenuated by HIF-1α activation.

### 3.4 Effects of anlotinib and bevacizumab on angiogenic signaling and the EMT in A549 and H1299 cells

**Figure 4:**
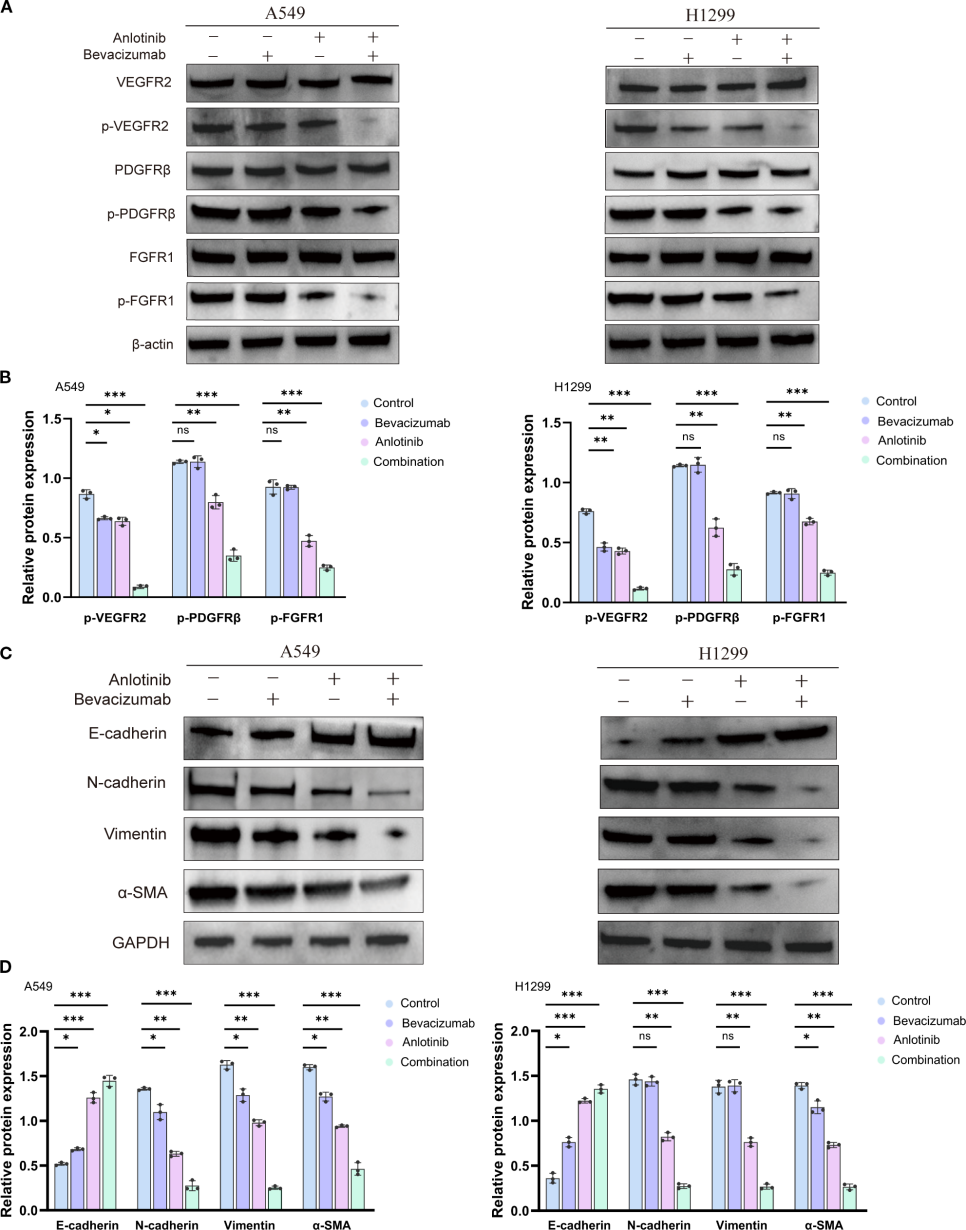
Effect of anlotinib and bevacizumab on angiogenesis and EMT in A549 and H1299 cells. **(A)** Western blotting of VEGFR2, PDGFRβ, FGFR1, p-VEGFR2, p-PDGFRβ, and p-FGFR1 protein in A549 and H1299 cells 24 h after treatment with 10 μM anlotinib, 600 μg/mL bevacizumab, or a combination of 10 μM anlotinib and 600 μg/mL bevacizumab for 24 h. **(B)** Quantitative analysis of blot shown in panel A for angiogenesis signaling proteins p-VEGFR2, p-PDGFRβ, and p-FGFR1 in A549 and H1299 cells. Protein levels were normalized to β-actin. **(C)** Western blotting of EMT marker proteins E-cadherin, N-cadherin, vimentin, and αSMA in A549 and H1299 cells treated with 10 μM anlotinib, 600 μg/mL bevacizumab, or a combination of 10 μM anlotinib and 600 μg/mL bevacizumab for 24 h. **(D)** Quantitative analysis of the blot shown in panel **(C)** Protein levels were normalized to GAPDH. Data are presented as mean ± SD for three independent experiments. ns = not significant; *p < 0.05, **p < 0.01, and ***p < 0.001. EMT, epithelial–mesenchymal transition.

Markers of angiogenesis, VEGFR2, PDGFRβ, FGFR1, and of the EMT, E-cadherin, N-cadherin, vimentin, and α-SMA, were assessed via Western blotting in A549 and H1299 cells. All three treatment regimens decreased the levels of phosphorylated angiogenesis markers p-VEGFR2, p-PDGFRβ, and p-FGFR1, but did not change the total protein levels of VEGFR2, PDGFRβ, and FGFR1 in both A549 and H1299 cells. Anlotinib had a greater impact than the two individual drug treatments, with combined therapy producing the most potent inhibition of any treatment (**Figures 4A, B**). These findings indicate that the anti-angiogenic effects of anlotinib, in particular, may be largely due to the inhibition of receptor phosphorylation. It may be that bevacizumab has the effect of increasing anlotinib targeting and that the enhanced anti-angiogenic effect of combined therapy may be due to the inhibition of multiple receptor tyrosine kinases.

In HUVECs, Western blotting and densitometric analysis revealed that bevacizumab or anlotinib alone produced minimal and statistically insignificant reductions in p-VEGFR2 and p-PDGFRβ levels compared with the control, whereas both agents modestly but significantly decreased p-FGFR1. The bevacizumab and anlotinib combination treatment markedly reduced the phosphorylation of VEGFR2, PDGFRβ, and FGFR1 to ~25%, ~25%, and ~20% of control values, respectively. The addition of HIF-1α inhibitor PX478 to the combination did not further alter phosphorylation, indicating that dual blockade had already achieved near-maximal inhibition. Conversely, HIF-1α activator DMOG partially reversed these inhibitory effects, restoring the phosphorylation of VEGFR2, PDGFRβ, and FGFR1 to ~80%, ~80%, and ~55% of control values, respectively (**Supplementary Figure S5**). These findings suggest that the anti-angiogenic efficacy of the combination treatment is closely linked to the suppression of HIF-1α and that HIF-1α reactivation mitigates receptor phosphorylation inhibition.

Western blotting analysis showed that individual treatment with anlotinib or bevacizumab reduced the expression of mesenchymal markers N-cadherin, vimentin, and α-SMA and increased epithelial marker E-cadherin relative to controls (**Figures 4C, D**). The lowest levels of N-cadherin, vimentin, and α-SMA and the highest level of E-cadherin were seen for the B+A combination therapy in both A549 and H1299 cells (**Figures 4C, D**). qRT–PCR also showed that the combination therapy suppressed the production of mRNA for mesenchymal markers and increased that for E-cadherin (**Supplementary Figures S6A, B**). Thus, the combination of anlotinib and bevacizumab appears to reverse the EMT in A549 and H1299 cells. Dual treatment with anlotinib and bevacizumab disrupted angiogenesis and the EMT, both of which processes are involved in tumor growth, metastasis, and drug resistance (40, 41). The combination therapy targets multiple signaling pathways to impede the formation of new blood vessels and reverse the phenotypic changes associated with EMT (42).

### 3.5 Anti-tumor effects of combined anlotinib and bevacizumab therapy {it}*in vivo*

**Figure 5:**
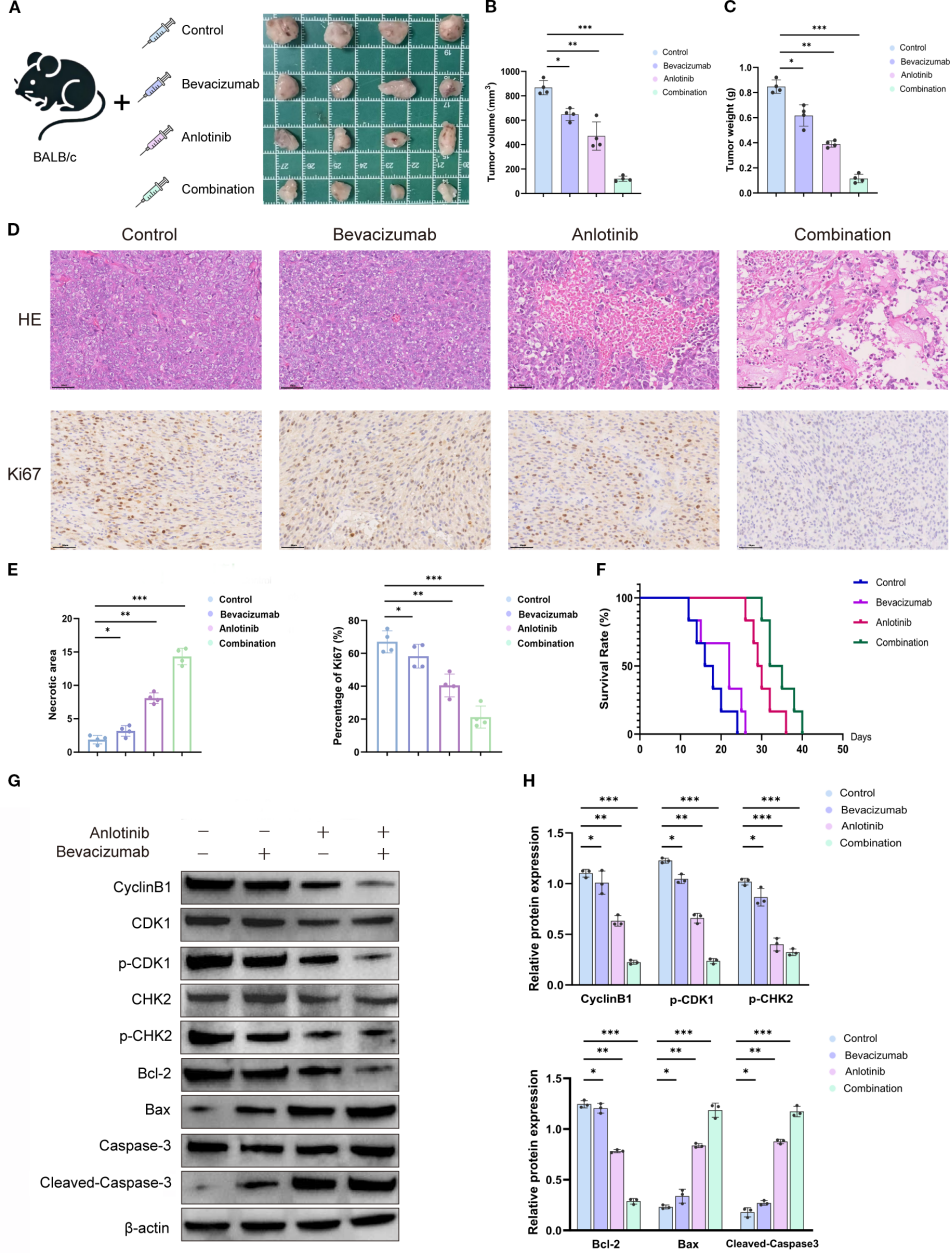
Effects of anlotinib, bevacizumab, and B+A combination treatment in a mouse xenograft model *in vivo*. **(A)** Tumor volume in the NSCLC xenograft model generated with A549 cells treated with 5 mg/kg bevacizumab twice weekly, 1 mg/kg anlotinib daily, or combination treatment with 5 mg/kg bevacizumab twice weekly and 1 mg/kg anlotinib daily. Representative tumor images after 28 days of treatment. **(B)** Quantitative analysis of tumor volume and weight after 40 days’ treatment (n = 4). **(C)** Tumor weight was measured at the end of the treatment. Combination therapy significantly decreased tumor weight compared to the control or single-agent treatments (n = 4 per group). **(D)** Histological analyses of tumors after 28 days’ treatment: H&E staining to show tumor morphology and IHC staining to show Ki-67 expression. **(E)** Quantitative analysis of tumor necrotic area and percentage of Ki-67+ cells. **(F)** Survival curves for mice with xenograft tumors treated with 5 mg/kg bevacizumab twice weekly, 1 mg/kg anlotinib daily, or combination treatment with 5 mg/kg bevacizumab twice weekly and 1 mg/kg anlotinib daily. **(G)** Western blotting of cell cycle markers cyclin B1, CDK1, p-CDK1, CHK2, and p-CHK2 and apoptosis markers Bcl2, Bax, caspase3, and cleaved caspase in tumor tissues. β-Actin was used as a loading control. **(H)** Quantitative analysis of blot shown in panel G normalized to β-actin expression. Scale bar = 250 μm. Data are presented as mean ± SD for three independent experiments. *p < 0.05, **p < 0.01, and ***p < 0.001. NSCLC, non-small cell lung cancer; IHC, immunohistochemical.

{/it}Xenograft tumors showed a 7.23-fold reduction in volume and a 7.08-fold reduction in weight following treatment of mice with both anlotinib and bevacizumab relative to controls. The inhibition of tumor growth is comparable with a 1.34-fold reduction in volume and a 1.37-fold reduction in weight for bevacizumab monotherapy and a 1.85-fold reduction in volume and a 2.18-fold reduction in weight for anlotinib monotherapy. These results illustrate the increased potency of the combined treatment (**Figures 5A–C**). H&E staining showed reduced tumor cellularity following the combination therapy, and measurements of Ki-67 expression showed a 68% reduction for B+A combined therapy, compared with a 13% reduction for bevacizumab monotherapy and a 39% reduction for anlotinib monotherapy (**Figures 5D, E**). The Kaplan–Meier survival analysis showed that the combined therapy extended mouse survival in comparison with controls or animals receiving monotherapy. The number of days at which 50% of treated animals survived was extended by 5 days for bevacizumab monotherapy, by 12 days for anlotinib monotherapy, and by 20 days for the combination of the two drugs relative to controls (**Figure 5F**).

Western blotting analyses of proteins involved in the cell cycle showed a downregulation of cyclin B1, p-CDK1, and p-CHK2 and unchanged levels of total CDK1 and CHK2, indicating cell cycle arrest after drug treatment (**Figure 5G**). Proapoptotic markers Bax and cleaved caspase-3 were increased, and antiapoptotic marker Bcl-2 was decreased, indicating increased apoptosis. Quantitative analyses indicated a 3.56–5.20-fold downregulation of the markers of the activated cell cycle and a 5.37-fold increase in apoptotic markers following the combination therapy (**Figure 5H**). The *in vivo* studies show that both anlotinib and bevacizumab monotherapies inhibited tumor growth by activating apoptosis and decreasing rates of cell proliferation. The combination of the two drugs dramatically enhanced the potency of these anti-tumor effects.

### 3.6 The effect of combination therapy on immune cell infiltration and vascular endothelial growth factor receptor activation

Immunohistochemical analysis was performed to assess immune cell infiltration and angiogenic receptor activation in tumor tissues from different treatment groups (**Supplementary Figure S7**). In the control group, the number of CD4^+^ T cells averaged approximately 65 per field. Bevacizumab monotherapy increased this slightly to approximately 70, without statistical significance, whereas anlotinib raised it to approximately 95, showing a significant increase compared with the control. The combination therapy produced the highest infiltration, reaching approximately 125 CD4^+^ T cells per field, significantly greater than that of both control and either monotherapy. A similar pattern was observed for CD8^+^ T cells. The numbers rose from approximately 50 in the control group to roughly 65 with bevacizumab and 80 with anlotinib. The combination treatment further enhanced CD8^+^ T-cell infiltration to approximately 105, significantly higher than that of all other groups. For angiogenic receptor phosphorylation, the combination therapy caused marked reductions. p-VEGFR2 expression decreased from roughly 95% positive area in the control group to 80% with bevacizumab and 65% with anlotinib, and further down to approximately 20% with the combination treatment. A similar trend occurred for p-PDGFRβ, which fell from approximately 90% in the control to 75% with bevacizumab and 65% with anlotinib, reaching approximately 15% with the combination treatment. For p-FGFR1, levels dropped from roughly 85% in the control to 70% with bevacizumab and 65% with anlotinib, and further to approximately 10% with the combination treatment. All reductions with the combination therapy were statistically significant. These results demonstrate that bevacizumab combined with anlotinib not only enhances intratumoral CD4^+^ and CD8^+^ T-cell infiltration but also strongly suppresses the activation of key angiogenic receptor tyrosine kinases, supporting its synergistic anti-angiogenic and immune-promoting effects.

**Figure 6:**
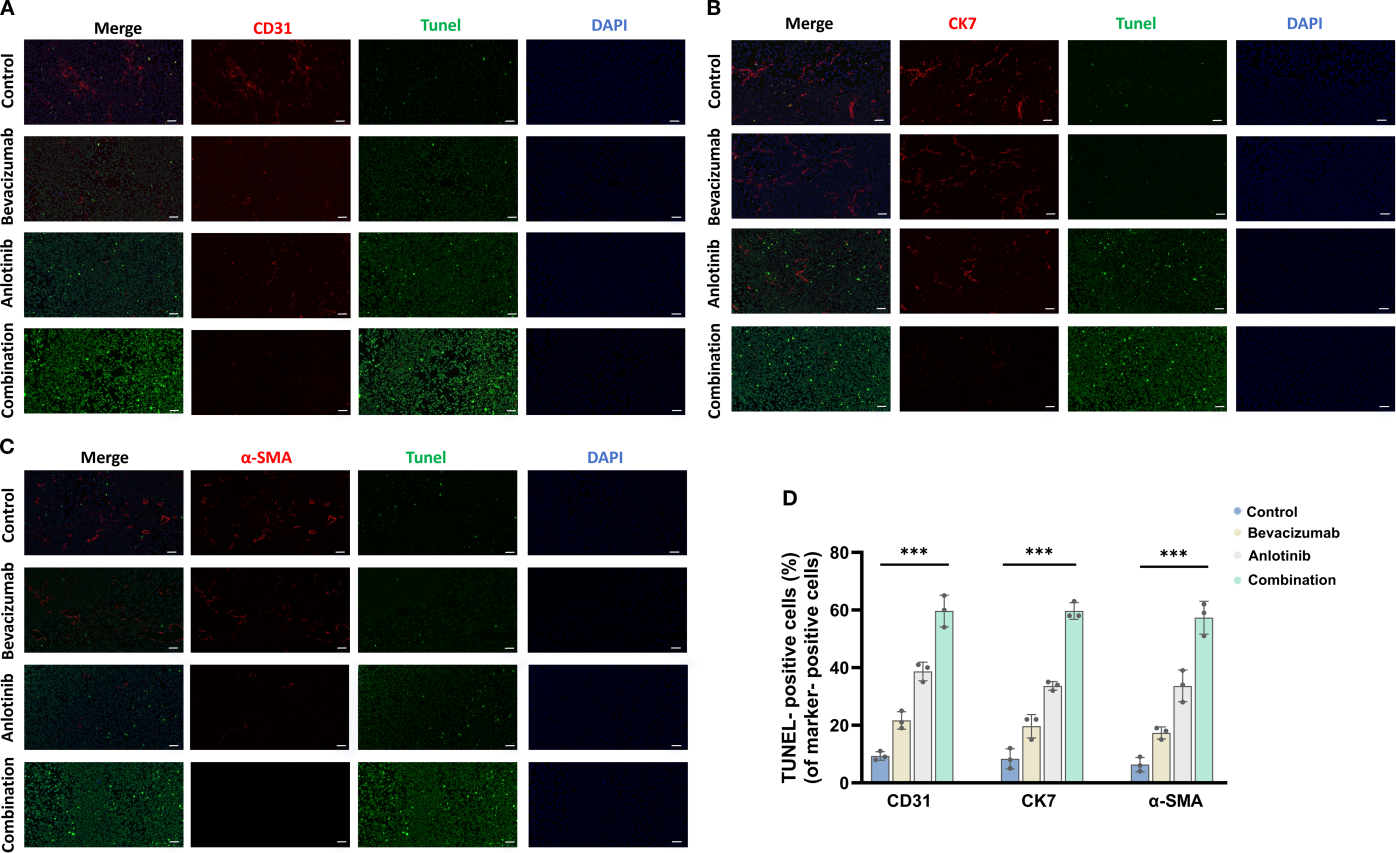
To assess apoptosis in distinct tumor cell populations, TUNEL staining was combined with markers for endothelial cells (CD31), epithelial/tumor cells (CK7), and fibroblasts/smooth muscle cells (α-SMA). **(A)** CD31, **(B)** CK7, **(C)** α-SMA, and **(D)** TUNEL-positive cells. Scale bar = 50 μm. Data are presented as mean ± SD for three independent experiments. ***p < 0.001.

To assess apoptosis in distinct tumor cell populations, TUNEL staining was combined with markers for endothelial cells (CD31), epithelial/tumor cells (CK7), and fibroblasts/smooth muscle cells (α-SMA). Quantitative analysis revealed that in CD31^+^ endothelial cells, the proportion of TUNEL-positive cells increased from approximately 10% in the control group to 20% with bevacizumab and 38% with anlotinib, reaching the highest level of approximately 60% in the combination group. A similar trend was observed for CK7^+^ epithelial/tumor cells, with TUNEL positivity rising from approximately 8% in the control group to 18% with bevacizumab and 35% with anlotinib, and further to approximately 58% with the combination treatment. For α-SMA^+^ fibroblasts/smooth muscle cells, the proportion of TUNEL-positive cells increased from approximately 7% in the control group to 15% with bevacizumab and 28% with anlotinib, reaching the highest level of approximately 57% in the combination group (**Figures 6A–D**). These results indicate that the combined bevacizumab and anlotinib treatment induces pronounced apoptosis across multiple tumor-associated cell types, with endothelial cells showing the highest apoptotic response.

### 3.7 Inhibitory effects of combined anlotinib and bevacizumab therapy on PI3K/AKT/HIF-1α signaling

**Figure 7:**
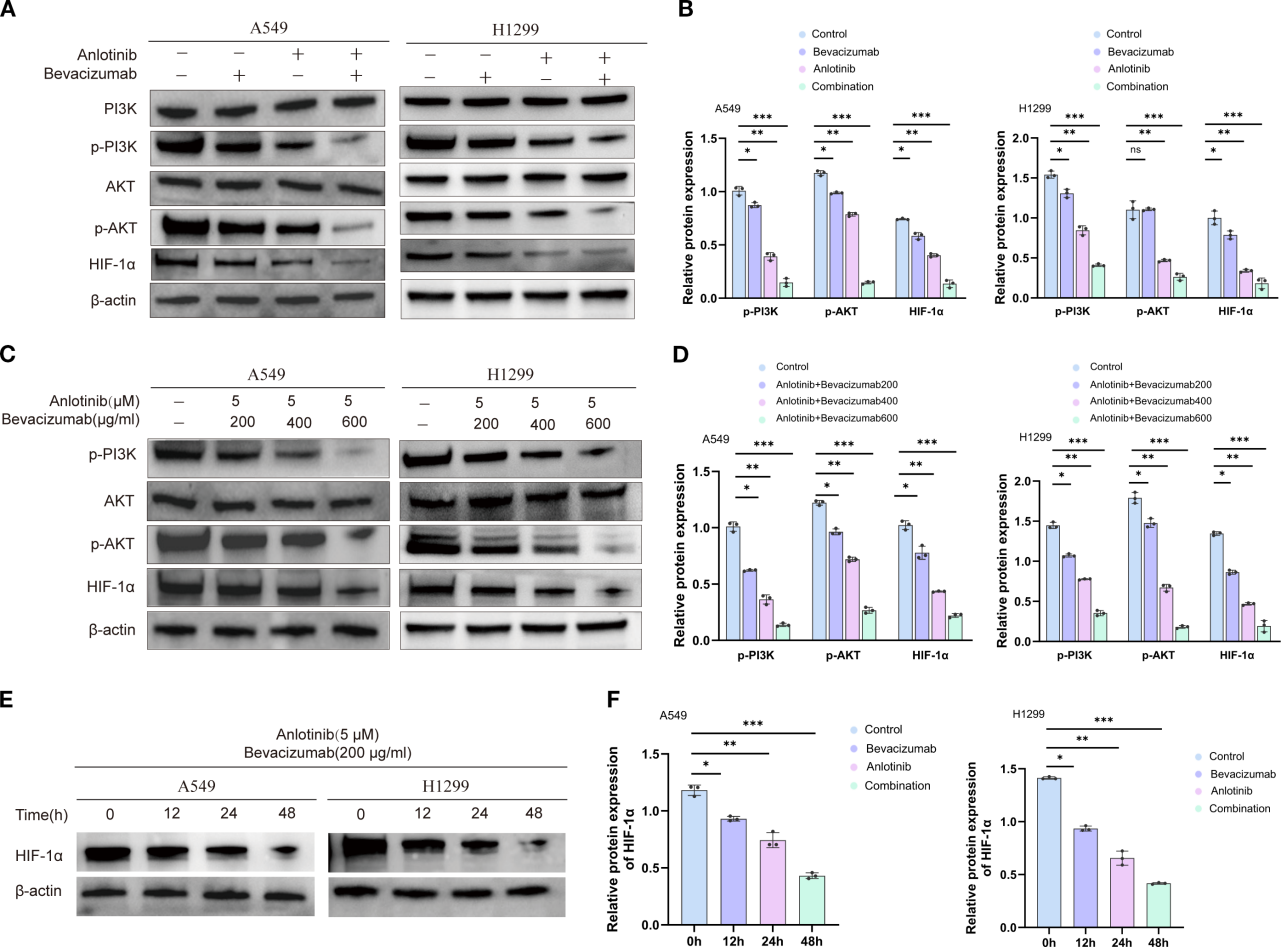
Effects of anlotinib, bevacizumab, and combination treatment on PI3K/AKT/HIF-1α signaling in A549 and H1299 cells. **(A)** Western blotting of markers of PI3K/AKT/HIF-1α signaling activation, p-PI3K, AKT, p-AKT, and HIF-1α, in A549 and H1299 cells after treatment with 10 μM anlotinib, 600 μg/mL bevacizumab, or a combination of 10 μM anlotinib and 600 μg/mL bevacizumab for 24 h. β-Actin was used as a loading control. **(B)** Quantitative analysis of blots shown in panel **(A, C)** Western blotting of p-PI3K, p-AKT, AKT, and HIF-1α showing the response to 24-h doses of 200, 400, and 600 μg/mL bevacizumab in combination with a fixed dose of 5 μM anlotinib in A549 and H1299 cells. β-Actin was used as a loading control. **(D)** Quantitative analysis of blots shown in panel **(C)** Levels were normalized to β-actin expression. **(E)** Western blotting of HIF-1α protein after treatment with 200 μg/mL bevacizumab in combination with a fixed dose of 5 μM anlotinib in A549 and H1299 cells for 0, 12, 24, and 48 h. β-Actin was used as a loading control. **(F)** Quantification of blot shown in panel **(E)**; HIF-1α protein was normalized to β-actin expression. Data are presented as mean ± SD for three independent experiments. ns = not significant; *p < 0.05, **p < 0.01, and ***p < 0.001.

Western blotting analysis of A549 and H1299 cells showed that 24 h of anlotinib treatment reduced the phosphorylation of PI3K and AKT (p-PI3K and p-AKT) by 33%–61% and downregulated HIF-1α expression by 63%, while total PI3K and AKT protein levels remained unchanged. Bevacizumab monotherapy also reduced p-PI3K and p-AKT by 13%–15% and HIF-1α by 21%. A more dramatic impact was found for the B+A combination therapy, which reduced the markers of PI3K signaling by approximately 80% and HIF-1α by 82% (**Figures 7A, B**). This suggests that this combination therapy approach is able to target the PI3K/AKT/HIF-1α pathway to a greater extent, thereby achieving an anti-tumor effect. Studies on dose dependence were conducted by holding the concentration of anlotinib at 5 μM while increasing the concentrations of bevacizumab from 200 to 400 and 600 μg/mL. A progressive reduction in p-PI3K, p-AKT, and HIF-1α levels was seen with increasing bevacizumab (**Figures 7C, D**). These findings support the view of a cooperative interaction between anlotinib and bevacizumab in targeting the PI3K/AKT/HIF-1α signaling pathway. Time-course experiments of HIF-1α expression at 0, 12, 24, and 48 h of treatment showed a gradual reduction in both cell types (**Figures 7E, F**). qRT–PCR analysis indicated a transcriptional downregulation of HIF-1α during drug treatment (**Supplementary Figure S8**). The data reported in this section show that anlotinib and bevacizumab inhibited PI3K/AKT/HIF-1α signaling in a dose-dependent manner and that HIF-1α was dynamically downregulated.

### 3.8 HIF-1α as an anti-tumor target of combined anlotinib and bevacizumab therapy

**Figure 8:**
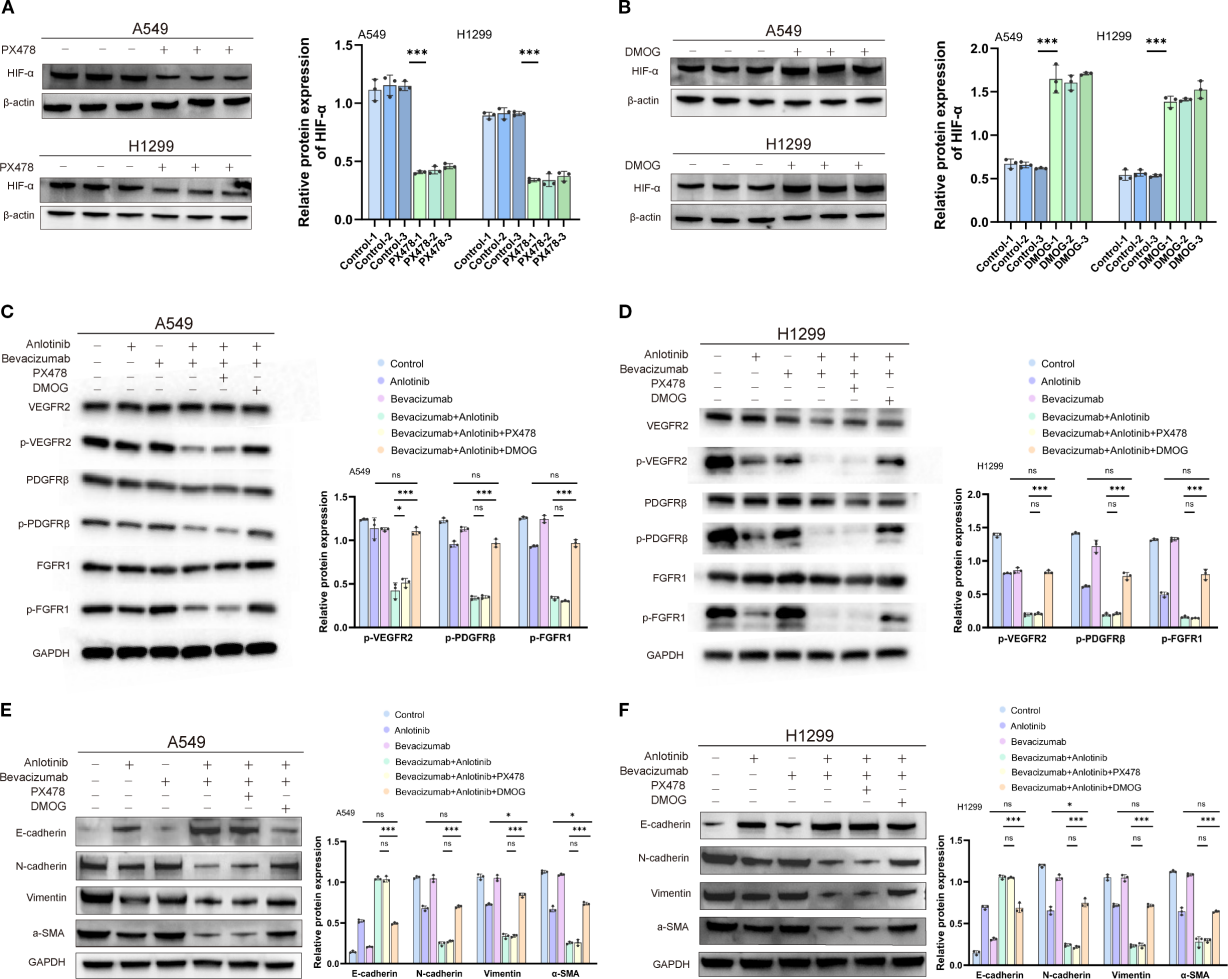
Effects of HIF-1α inhibitor PX478 and activator DMOG on expression of HIF-1α, markers of angiogenesis, and markers of the EMT in A549 and H1299 cells treated with anlotinib, bevacizumab, or B+A combination therapy. **(A)** Western blotting image (left panel) of HIF-1α protein in A549 and H1299 cells after 12-h treatment with 5 µM PX478. β-Actin was used as a loading control. Quantitative analysis (right panel) of HIF-1α expression normalized to β-actin. **(B)** Western blotting image (left panel) of HIF-1α protein in A549 and H1299 cells after 12-h treatment with 200 µM DMOG. β-Actin was used as a loading control. Quantitative analysis (right panel) of HIF-1α expression normalized to β-actin. **(C)** Western blotting images (left panel) of phosphorylated and total VEGFR2, PDGFRβ, and FGFR1 in A549 cells after treatment with 10 μM anlotinib, 600 μg/mL bevacizumab, or a combination of 10 μM anlotinib and 600 μg/mL bevacizumab or combination therapy with 5 µM PX478 or 200 µM DMOG for 24 h. GAPDH was used as a loading control. Quantitative analysis normalized to GAPDH (right panel). **(D)** Western blotting images (left panel) of phosphorylated and total VEGFR2, PDGFRβ, and FGFR1 in H1299 cells after treatment with 10 μM anlotinib, 600 μg/mL bevacizumab, or a combination of 10 μM anlotinib and 600 μg/mL bevacizumab or combination therapy with PX478 or DMOG for 24 h. GAPDH was used as a loading control. Quantitative analysis normalized to GAPDH (right panel). **(E)** Western blotting image (left panel) of EMT markers E-cadherin, N-cadherin, vimentin, and α-SMA in A549 cells after treatment with 10 μM anlotinib, 600 μg/mL bevacizumab, or a combination of 10 μM anlotinib and 600 μg/mL bevacizumab or combination therapy with PX478 or DMOG for 24 h. GAPDH was used as a loading control. Quantitative analysis (right panel) normalized to GAPDH. **(F)** Western blotting image (left panel) of EMT markers E-cadherin, N-cadherin, vimentin, and α-SMA in H1299 cells after treatment with 10 μM anlotinib, 600 μg/mL bevacizumab, or a combination of 10 μM anlotinib and 600 μg/mL bevacizumab or combination therapy with PX478 or DMOG for 24 h. GAPDH was used as a loading control. Quantitative analysis (right panel) normalized to GAPDH. Data are presented as mean ± SD for three independent experiments. ns = not significant; *p < 0.05 and ***p < 0.001. EMT, epithelial–mesenchymal transition.

Treatment of A549 and H1299 cells with HIF-1α inhibitor PX478 suppressed HIF-1α protein, whereas treatment with HIF-1α activator DMOG stimulated HIF-1α expression compared with the controls (**Figures 8A, B**). The reduced phosphorylation of angiogenesis markers VEGFR2, PDGFRβ, and FGFR1 was reported above in response to combined anlotinib and bevacizumab therapy. The addition of PX478 did not further enhance these anti-angiogenic effects, while the addition of DMOG restored the phosphorylation levels to those observed with anlotinib monotherapy, effectively neutralizing the contribution of bevacizumab in the combination therapy (**Figures 8C, D**). This finding is consistent with the view that the impact of bevacizumab depends on HIF-1α suppression. It was also reported above that combined therapy with anlotinib and bevacizumab increased epithelial marker E-cadherin and reduced mesenchymal markers N-cadherin, vimentin, and α-SMA, indicating the inhibition of the EMT. The addition of PX478 did not amplify these changes, and the addition of DMOG restored EMT marker levels to those observed with anlotinib monotherapy (**Figures 8E, F**). These findings are consistent with the view that the anti-EMT effects of the combination therapy are mediated through HIF-1α suppression.

**Figure 9:**
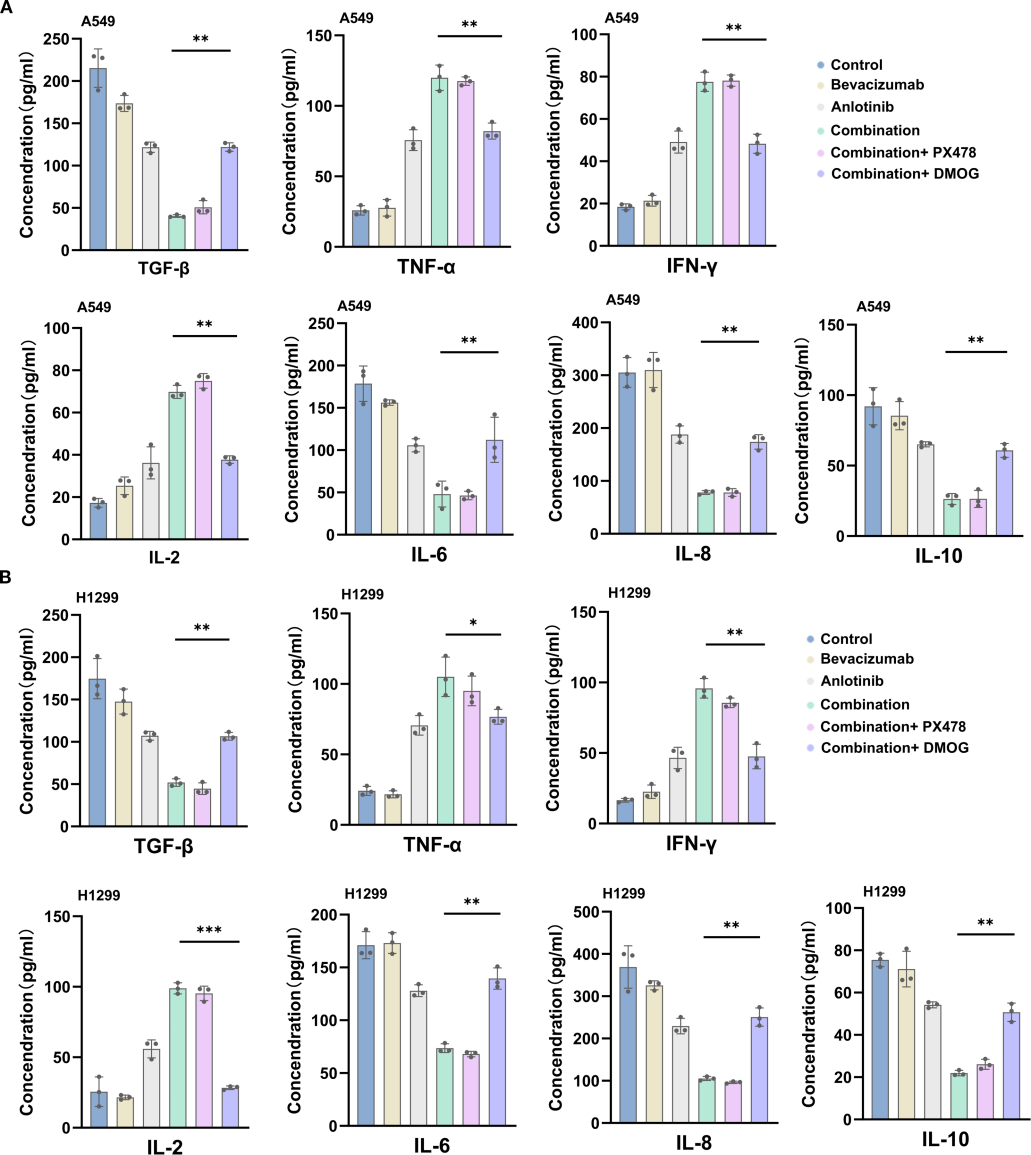
ELISA detection of pro-angiogenic/immunosuppressive cytokine levels after combination therapy with bevacizumab and anlotinib. **(A)** A549 cell. **(B)** H1299 cell. Data are presented as mean ± SD for three independent experiments. ns = not significant; *p < 0.05, **p < 0.01, and ***p < 0.001.

ELISA showed that in both A549 and H1299 cells, TGF-β was the highest in the control group (212 and 193 pg/mL), decreased in the bevacizumab group (175 and 152 pg/mL) and the anlotinib group (124 and 103 pg/mL), and was the lowest in the combination group (52 and 48 pg/mL). TNF-α increased from 42 and 37 pg/mL in controls to 108 and 112 pg/mL in the combination group, IFN-γ from 19 and 18 to 80 and 91 pg/mL, and IL-2 from 21 and 27 to 75 and 101 pg/mL. In contrast, IL-6 decreased from 322 and 175 pg/mL in controls to 95 and 54 pg/mL in the combination group, IL-8 from 558 and 445 to 142 and 142 pg/mL, and IL-10 from 90 and 74 to 29 and 23 pg/mL. Adding PX478 to the combination yielded values close to those of the combination group, whereas DMOG partially reversed these effects, increasing TGF-β to 116 and 95 pg/mL, IL-6 to 182 and 127 pg/mL, and IL-8 to 256 and 214 pg/mL (**Figures 9A, B**). Overall, bevacizumab plus anlotinib markedly reduced pro-angiogenic/immunosuppressive cytokines (TGF-β, IL-6, IL-8, and IL-10) and increased pro-inflammatory cytokines (TNF-α, IFN-γ, and IL-2) in both NSCLC cell lines, with these effects being at least partly dependent on HIF-1α signaling.

Ang2, GLUT1, LDHA, and PDK1 are known to be downstream targets of HIF-1α. The combination therapy suppressed the expression of these proteins, indicating reduced HIF-1α-mediated metabolic signaling in addition to the previously noted reduced angiogenic signaling. The addition of PX478 did not produce any further suppression of Ang2, GLUT1, LDHA, and PDK1 expression; the addition of DMOG restored levels comparable to those observed with anlotinib monotherapy, neutralizing the contribution of bevacizumab to the combination therapy (**Supplementary Figures S9A, B**). qRT–PCR expression analyses produced consistent results (**Supplementary Figures S10**, **S11**). In summary, HIF-1α is instrumental in the anti-tumor effects of anlotinib and bevacizumab and appears to account for the contribution made by the latter to the combination therapy. Reduced HIF-1α activity inhibits angiogenic signaling pathways and reverses the EMT (43, 44).

## 4 Discussion

A vascularized microfluidic chip platform to recapitulate the formation of functional, interconnected vascular networks was developed and presented. The microfluidic platform promotes 3D tissue formation and dynamic interactions through continuous perfusion (45, 46), more accurately simulating the *in vivo* environment than 2D cell culture, and represents an *in vitro* model for the study of angiogenesis and the assessment of antiangiogenic drugs. We suggest that the microfluidic platform is suitable for high-throughput drug screening and mechanistic studies. The utilization of the novel platform allowed the demonstration that anlotinib and bevacizumab disrupted vascular integrity and decreased vascular permeability, effects that were more pronounced for the combination of the two drugs than for either monotherapy (37, 47).

The antiangiogenic activities of anlotinib (which targets receptor tyrosine kinases) and bevacizumab (which inhibits VEGF-A) have led to their being drugs of choice for many cancers, including NSCLC (48, 49). However, anti-tumor monotherapy is often hindered by drug resistance (49, 50), and this is particularly true for bevacizumab (50, 51). There have been suggestions that the increase in T-cell infiltration elicited by anlotinib enhances the anti-tumor immune response and may contribute to reversing *in vivo* drug resistance (52). These observations make the combination of anlotinib and bevacizumab an attractive proposition for cancer treatment. The current findings support such a view with bevacizumab targeting HIF-1α to reduce the progression of tumor development, while anlotinib reinforces these actions and may also protect the body against drug resistance. The use of HIF-1α inhibitor PX478 and activator DMOG indicated that the targeting of angiogenesis and EMT markers was due to the impact of bevacizumab in suppressing HIF-1α activity. In addition, using HUVEC scratch wound and Transwell invasion assays, we found that the combination therapy produced a markedly greater inhibition of cell migration and invasion than either monotherapy. Importantly, these effects were largely unaffected by HIF-1α inhibitor PX478 but were partially reversed by HIF-1α activator DMOG, suggesting that the suppression of HIF-1α signaling plays a central role in mediating the observed anti-migratory and anti-invasive activities. HIF-1α is known to be a master regulator of tumorigenesis, altering the transcription of many genes involved in cell proliferation and bringing about the metabolic adaptations that characterize tumor cells (53). The current work found that the downstream targets of HIF-1α activity, metabolic markers Ang2, GLUT1, LDHA, and PDK1, which equip the tumor for survival and growth under hypoxic conditions (43, 44), were also downregulated by anlotinib plus bevacizumab. It is interesting to note that the use of PX478 and DMOG indicates that this downregulation is largely, if not entirely, produced by the actions of bevacizumab.

PI3K/AKT/HIF-1α signaling has been previously found to be involved in angiogenesis, metabolism, survival, and the EMT (54, 55). Preclinical studies have shown that HIF-1α inhibitors exert anti-tumor effects by inhibiting genes related to glycolysis, angiogenesis, and metastasis in tumor cells. For example, PX478 significantly reduced HIF-1α levels and inhibited tumor growth in a lung cancer xenograft model, and BAY 87–2243 restored the balance of CD4+ T-cell subsets and reduced the production of the pro-inflammatory cytokines, thus acting as both an immune imbalance regulator and anti-inflammatory (56, 57). The utility of drug combinations has been increasingly recognized, including HIF-1α inhibitors plus immune checkpoint inhibitors. Such therapeutic strategies aim to overcome microenvironment hypoxia-mediated immune suppression by multi-pathway targeting (58). Previous studies in this area have given reference points for HIF-1α-based combination therapy, and the B+A combination regimen used in the present study compares favorably and may contribute to overcoming the limitations of single agents.

HIF-1α is shown to influence angiogenesis and the EMT in NSCLC, and it is also clear that the bevacizumab component is largely responsible for the inhibitory effect on these processes of B+A treatment. The inhibition of HIF-1α has previously been shown to reduce tumor-induced immune suppression (59, 60), and B+A combination therapy has the potential to reprogram the tumor microenvironment and facilitate anti-tumor immune responses. Previous studies have shown that bevacizumab inhibits VEGF signaling and reduces immunosuppressive cells and that multitarget TKIs block the VEGFR/PDGFR/FGFR pathway, promoting vascular normalization and alleviating tumor immune escape (16, 50, 51, 61). The B+A combination inhibited the HIF-1α/PI3K/AKT axis and reduced the levels of pro-angiogenic factors. The B+A strategy may synergistically enhance T/NK cell infiltration and activation through a dual mechanism involving the inhibition of abnormal angiogenesis and the alleviation of the hypoxic microenvironment. Single-cell sequencing or spatial multi-omics may give further information regarding the dynamic regulation of the tumor immune landscape by the combination therapy, facilitating clinical translation. The potential for clinical use is promising. First, the two drugs target VEGF signaling (bevacizumab) and multi-kinase pathways (anlotinib), leading to blockade of tumor angiogenesis and reducing compensatory drug resistance caused by single target inhibition. Second, the inhibition of hypoxia mediator HIF-1α suggests suitability for the treatment of highly vascularized or metastatic lesions in advanced NSCLC. Future sequential or synergistic combination therapy plus immune checkpoint inhibitors may be optimized by dynamic HIF-1α activity monitoring.

In this study, monotherapy with either bevacizumab or anlotinib had only modest effects on the phosphorylation of VEGFR2, PDGFRβ, and FGFR1, whereas the combination treatment reduced phosphorylation levels to 20%–30% of controls. This indicates that the simultaneous blockade of VEGF ligand binding and multiple angiogenic receptor tyrosine kinases produces a more complete inhibition of angiogenic signaling cascades. The partial restoration of receptor phosphorylation by DMOG further supports a mechanistic link between HIF-1α activation and downstream RTK reactivation, consistent with the role of HIF-1α in regulating pro-angiogenic gene expression under hypoxic conditions. In addition, angiogenic markers were inhibited at the functional level (i.e., phosphorylation), whereas EMT markers were fully regulated at the expression level, reflecting the synergistic anti-tumor effects of anlotinib in combination with bevacizumab treatment through multiple mechanisms and levels working together. This in-depth understanding of the differential protein expression will help to elucidate the mechanism of action of the combination therapy and provide a theoretical basis for the further optimization of targeted therapeutic strategies for NSCLC. In terms of angiogenic signaling, when HIF-1α expression was activated, its downstream pro-angiogenic pathway was reactivated, thus counteracting the pathway of action of bevacizumab by “depleting VEGF”. Thus, this “expression restoration” phenomenon reveals a mechanistic focus: HIF-1α serves as a bridge between the receptor inhibitory effects of anlotinib and the ligand antagonistic effects of bevacizumab, and the synergistic effect of both is dependent on the effective inhibition of HIF-1α. Overall, the action of bevacizumab in combination with anlotinib is dependent on the sustained inhibition of HIF-1α, and its regulation of angiogenesis, EMT, and metabolic pathways is all reflected in the differential regulation of HIF-1α-related protein expression. This differential protein expression reflects the dependence and complementarity between different drug mechanisms and provides theoretical support for the subsequent development of combination strategies targeting HIF-1α.

Studies have shown that VEGF-driven tumor vascular abnormalities not only support tumor growth but also aid in tumor immune escape by promoting an immunosuppressive microenvironment (62). Anti-angiogenic therapy can enhance the efficacy of immune checkpoint inhibitors by temporarily normalizing the vasculature, alleviating hypoxia, and improving immune cell infiltration and function (63). Several clinical trials are currently exploring this combination strategy, such as anlotinib in combination with PD-1 inhibitors in pancreatic cancer (64) and bevacizumab in combination with atezolizumab in cervical cancer (65). The results of this study showed that the combination therapy not only suppressed angiogenic receptor activation but also enhanced anti-tumor immunity. CD4^+^ and CD8^+^ T-cell infiltration was substantially increased in the combination group, indicating a shift toward a more immunologically active tumor microenvironment. This aligns with the concept that vascular normalization induced by anti-angiogenic therapy can improve immune cell trafficking into tumors.

The modulation of immune parameters observed here further supports a broader impact on the tumor microenvironment. Enhanced infiltration of CD4^+^ and CD8^+^ T cells, along with increased TNF-α, IFN-γ, and IL-2, suggests improved immune activation, possibly through vascular normalization and reduced immunosuppressive cytokines like TGF-β, IL-6, IL-8, and IL-10. This shift toward a pro-inflammatory milieu could sensitize tumors to immune checkpoint blockade, aligning with growing evidence that effective anti-angiogenic therapy can synergize with immunotherapy. Clinically, these results point toward a therapeutic rationale for combining bevacizumab and anlotinib in advanced NSCLC, particularly in patient subsets characterized by high HIF-1α expression or resistance to prior VEGF-targeted therapy. The ability of this regimen to concurrently disrupt angiogenesis, enhance anti-tumor immunity, and induce apoptosis across multiple tumor-associated cell types could yield more durable tumor control. Prospective studies integrating this combination with immunotherapeutic agents and biomarker-driven patient selection would help clarify its full clinical potential.

Although this study mainly focused on elucidating the anti-angiogenic effect of this combination therapy through a vascularized microfluidic microarray model and *in vitro* experiments, we also recognize that further comprehensive validation *in vivo* will help to enhance the persuasiveness of the study findings. In subsequent studies, we plan to conduct more systematic *in vivo* experiments, including angiogenesis-related indices as well as immune cell infiltration-related markers, in order to more comprehensively assess the effects of the combination therapy on the tumor microenvironment. In addition, the use of an immunologically intact mouse model will help to more accurately assess its potential immunomodulatory effects, which will also be an important direction for our subsequent studies. The findings of this study provide a solid foundation for further exploration of this combination therapy strategy.

We acknowledge some limitations to the current study. First, the microfluidic chip model lacks the complexity of the tumor microenvironment and allows only limited assessment of cellular interactions. Chip design may be optimized in the future to allow the inclusion of stromal cells, such as those from the immune system. Second, the current *in vivo* model included only one type of NSCLC, and future work may address different subtypes and examine cancer treatment in a clinical setting. Third, the present work focused on PI3K/AKT/HIF-1α signaling, but other pathways may also be involved in the responses to anlotinib and bevacizumab. Genomic and proteomic analyses may indicate additional targets and resistance mechanisms and reveal interactions between HIF-1α and other pathways.

## 5 Conclusion

A vascularized microfluidic chip platform was constructed and used to test the efficacy of the anti-angiogenic drugs anlotinib and bevacizumab. Anlotinib and bevacizumab were shown to have a synergistic impact on reducing the growth of NSCLC cells *in vitro* and the growth of NSCLC xenograft tumors *in vivo*. The two drugs inhibited tumor cell proliferation by stimulating apoptosis, reducing angiogenesis, and reversing the EMT. The most potent effect was seen for the combination of anlotinib and bevacizumab. The suppression of tumor cell master regulator HIF-1α was involved in the drug effects. Importantly, the combination therapy enhanced the infiltration of T cells, increased the levels of pro-inflammatory cytokines, and simultaneously alleviated immunosuppression, thereby enhancing anti-tumor immunity. These findings illustrate the value of the microfluidic chip platform for drug testing and highlight the therapeutic promise of combining anlotinib and bevacizumab in NSCLC. This strategy provides a theoretical basis for overcoming the resistance of single therapy and lays the foundation for its future combination with immunotherapy in clinical settings.

## Data Availability

The datasets presented in this study can be found in online repositories. The names of the repository/repositories and accession number(s) can be found below: https://doi.org/10.6084/m9.figshare.28876859.
