# Supplementary material for: Bevacizumab and anlotinib combination therapy acts via HIF-1α suppression to exert synergistic anti-angiogenic and anti-tumor effects in non-small cell lung cancer
Source: Front Immunol. 2025 Sep 17;16:1613368. doi: 10.3389/fimmu.2025.1613368 (PMC12484198; doi:10.3389/fimmu.2025.1613368)
Supplement: Supplementary file 2 [file DataSheet2.pdf]

广州水云天生物科技有限公司

实验动物福利与伦理审查同意书

编号：SYT2024084

南方医科大学 娜飞沙·斯马义 研究组

你组 娜飞沙·斯马义 申请“安罗替尼联合贝伐珠单抗通过PI3K/AKT/HIF-1a通路协同抗肿瘤作用的研究”项目拟开展的动物实验：SYT2024084，经实验动物福利与伦理委员会审核并通过，相关动物实验符合福利与伦理原则。申请人及各参与人须按照动物实验相关规定及福利与伦理要求严格实施该项动物实验，预祝实验取得理想结果。

实验动物福利与伦理委员会(盖章)

2024年01月02日

Animal Welfare and Ethics Review Consent Form

Number：SYT2024084

Southern Medical University

The research team, including Nafeisha Simayi, and others, has applied for the project titled "Study on the Synergistic Antitumor Effect of Anlotinib Combined with Bevacizumab through the PI3K/AKT/HIF-1a Pathway" (SYT2024084). This project’s animal experiments have been reviewed and approved by the Animal Welfare and Ethics Committee, ensuring that the experiments adhere to animal welfare and ethical principles.The applicant and all involved personnel must strictly comply with the relevant animal experimental regulations and welfare and ethics requirements in the implementation of this project. We wish the experiment successful results.

Animal Welfare and Ethics Review Committee

January 2, 2024
