# Supplementary material for: Bevacizumab and anlotinib combination therapy acts via HIF-1α suppression to exert synergistic anti-angiogenic and anti-tumor effects in non-small cell lung cancer
Source: Front Immunol. 2025 Sep 17;16:1613368. doi: 10.3389/fimmu.2025.1613368 (PMC12484198; doi:10.3389/fimmu.2025.1613368)
Supplement: Supplementary file 3 [file Table1.docx]

# **Supplementary materials**

**Table S1**. Primer sequences for qRT-PCR analysis.

| Gene Name | Forward Primer (5'-3') | Reverse Primer (5'-3') |
| --- | --- | --- |
| E-Cadherin | CCTTCCTCCCAATACATCTCC | GGTCGTTGTACTGAATGGTCC |
| N-Cadherin | ATATGCCCAAGACAAAGAGAC | CTTCAACTTCTGCTGACTCCT |
| Vimentin | GACGCCATCAACACCGAGTT | TTTGTCGTTGGTTAGCTGGT |
| α-SMA | GGCATTCACGAGACCACCTAC | CGACATGACGTTGTTGGCATAC |
| HIF-1α | CGGAGTGAGGAGGAAGAGG | GGAAGTGGAGGGGAAGAGG |
| Ang2 | GCAGGAGCGGGCTGGACTG | GCACAGGTGCTGTGGATGAG |
| GLUT1 | GCCGAGGGAGAGAGGAAGG | GGAGGAGGGGAGGGAGAGG |
| LDHA | CGCGGAGGTGGAGGAGGAG | CCAGGAAGAGGAGGAGAGGA |
| PDK1 | GTGCGAGGTGGAGGAAGGA | AGGGAGGAGGGAGGAGGAGG |
| GAPDH | TGCACCACCAACTGCTTAG | GGCATGGACTGTGGTCATG |

**Table S2.** List of primary antibodies.

| Protein | Manufacturer | Dilution Ratio |
| --- | --- | --- |
| VEGFR2 | Cell Signaling Technology | 1:500 |
| p-VEGFR2 | Cell Signaling Technology | 1:500 |
| PDGFRβ | Cell Signaling Technology | 1:500 |
| p-PDGFRβ | Cell Signaling Technology | 1:500 |
| p-PDGFRβ | Cell Signaling Technology | 1:500 |
| FGFR1 | Cell Signaling Technology | 1:500 |
| p-FGFR1 | Cell Signaling Technology | 1:500 |
| E-cadherin | Abcam | 1:1000 |
| N-cadherin | Abcam | 1:1000 |
| Vimentin | Abcam | 1:1000 |
| α-SMA | Abcam | 1:1000 |
| PI3K | Cell Signaling Technology | 1:800 |
| p-PI3K | Cell Signaling Technology | 1:800 |
| AKT | Cell Signaling Technology | 1:800 |
| p-AKT | Cell Signaling Technology | 1:800 |
| HIF-1α | Cell Signaling Technology | 1:800 |
| Cyclin B1 | Cell Signaling Technology | 1:1000 |
| CDK1 | Abcam | 1:1000 |
| p-CDK1 | Abcam | 1:1000 |
| CHK2 | Cell Signaling Technology | 1:1000 |
| p-CHK2 | Cell Signaling Technology | 1:1000 |
| Bax | Cell Signaling Technology | 1:1000 |
| Bcl-2 | Cell Signaling Technology | 1:1000 |
| Caspase-3 | Cell Signaling Technology | 1:1000 |
| Cleaved-Caspase3 | Cell Signaling Technology | 1:1000 |
| Ang2 | Abcam | 1:1000 |
| GLUT1 | Abcam | 1:1000 |
| LDHA | Abcam | 1:1000 |
| PDK1 | Abcam | 1:1000 |
| GAPDH | Cell Signaling Technology | 1:1000 |
| β-actin | Cell Signaling Technology | 1:1000 |

**
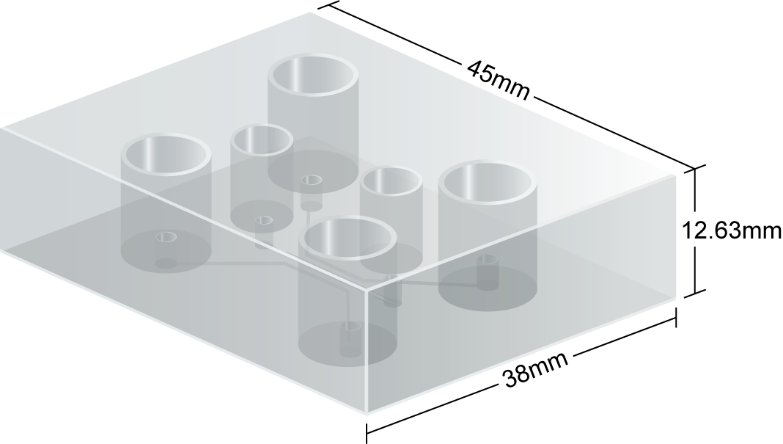
**

**Figure S1**. 3D perspective diagram of the microfluidic chip, showing layout and dimensions (45 mm × 38 mm × 12.63 mm).


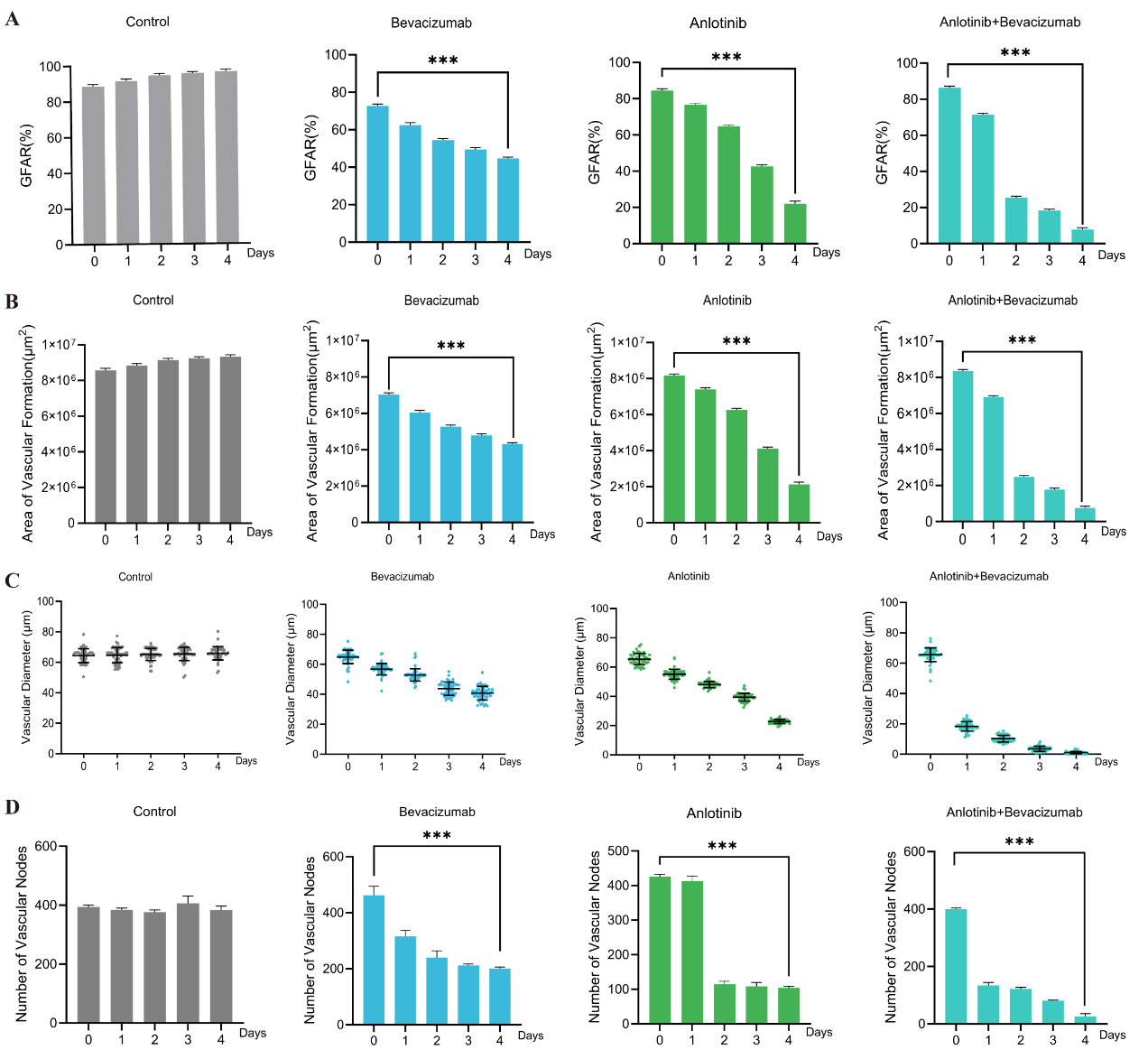


**Figure S2**. A GFP area ratio (GFAR) over time for different treatment groups; B. Area of Vascular formation relative to controls over time; C. Temporal changes in vascular diameter for different treatment groups; D. Temporal changes in vascular node count for different treatment groups. Data are presented as mean ± SD from three independent experiments. ns = not significant, ****p* < 0.001.

**
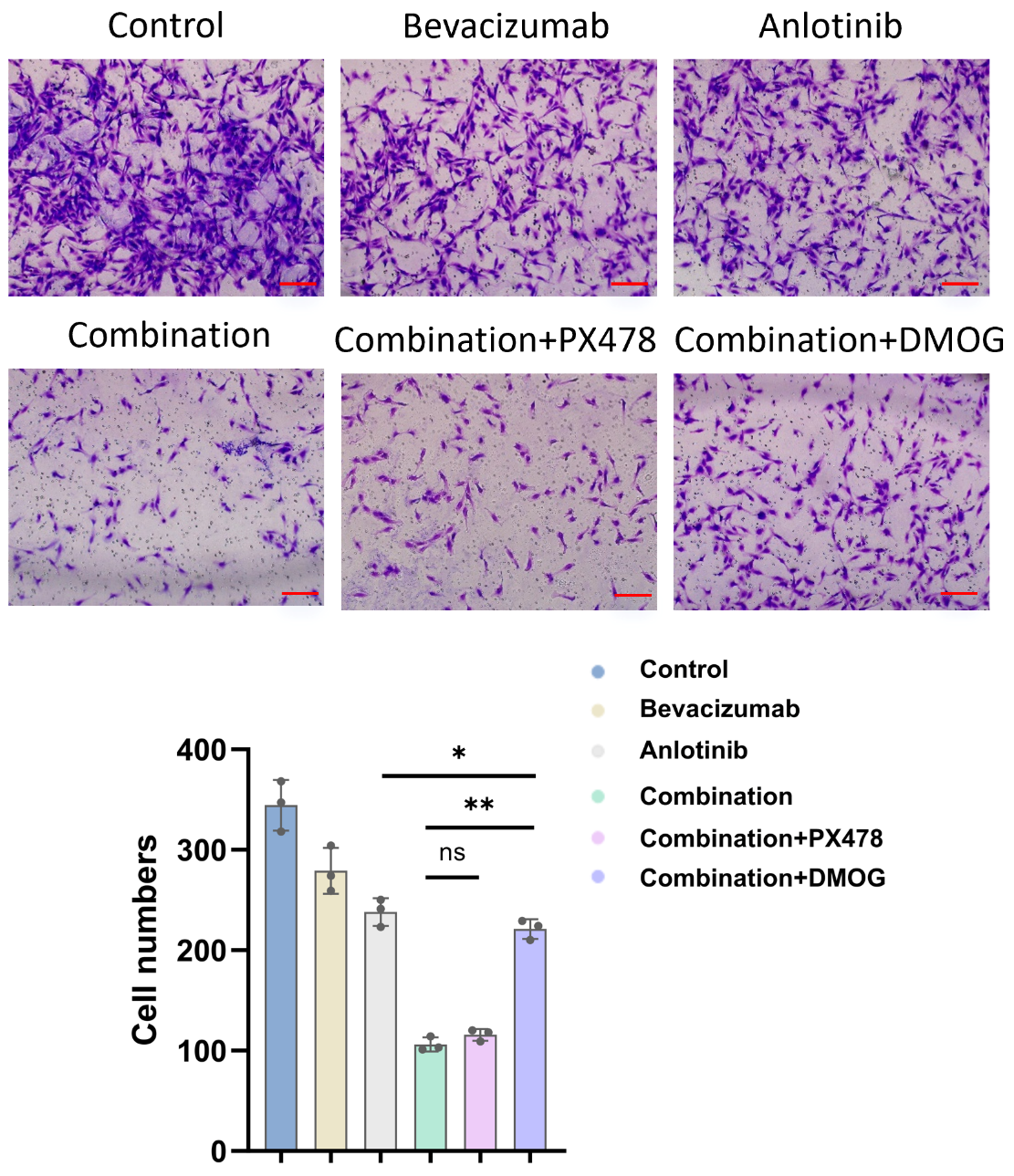
**

**Figure S3. The invasive capacity of HUVECs was evaluated using the Transwell invasion assay.** Scale bar = 150μm. Data are presented as mean ± SD from three independent experiments. ns = not significant, **p* < 0.05, ***p* < 0.01.

**
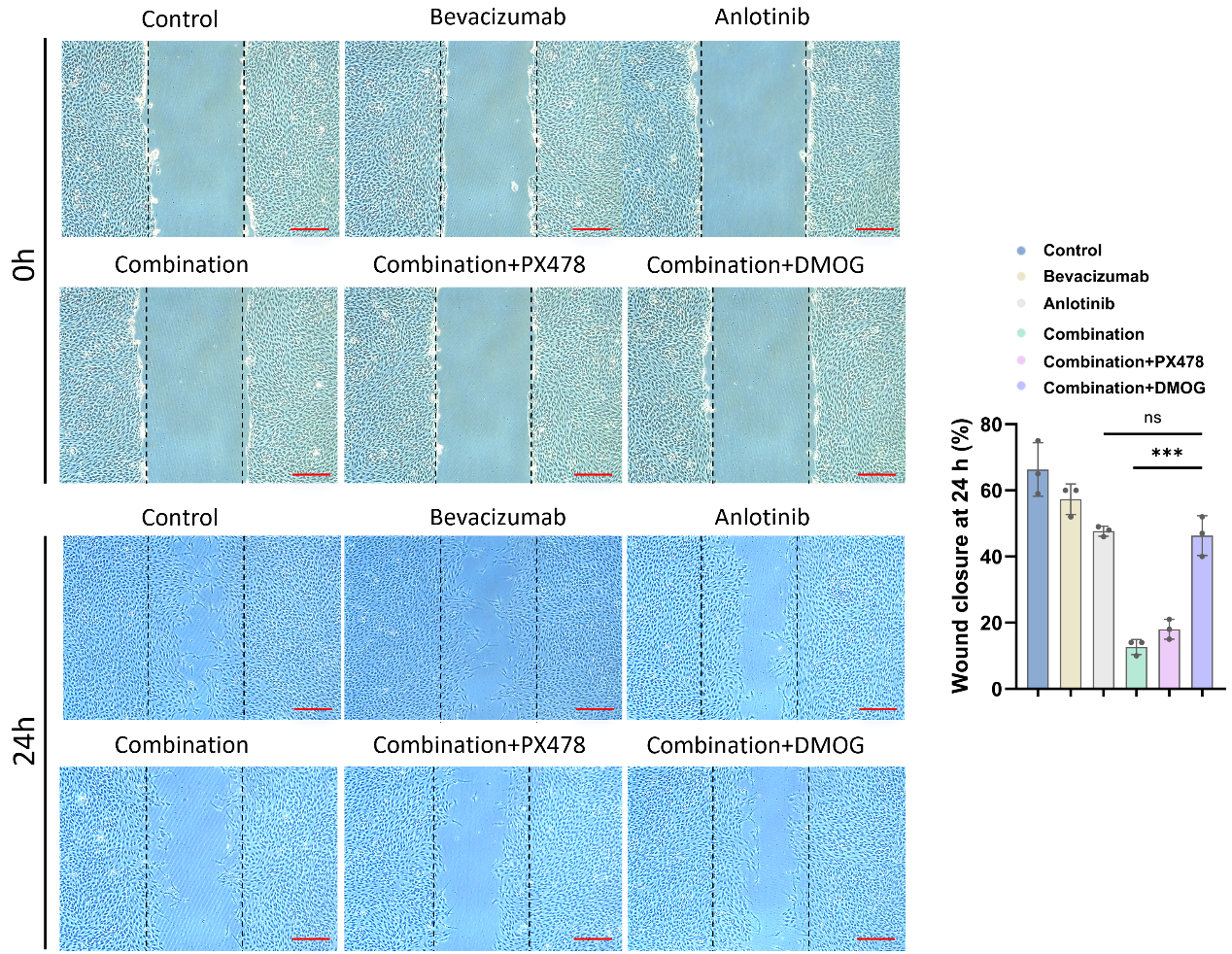
**

**Figure S4.** The effect of the treatments on HUVEC migration was evaluated using a wound healing assay. Scale bar = 500μm. Data are presented as mean ± SD from three independent experiments. ns = not significant, ****p* < 0.001.

**
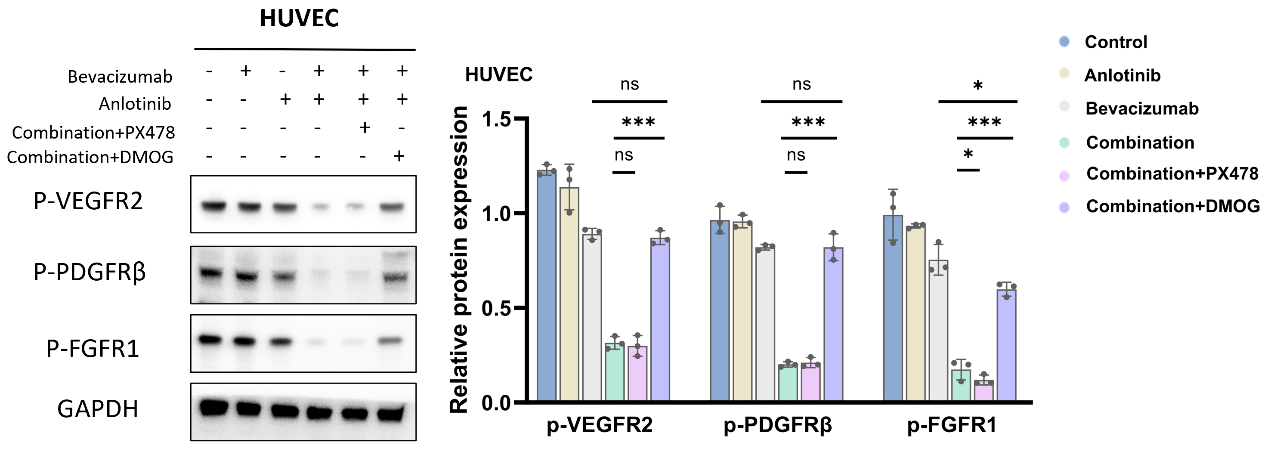
**

**Figure S5.** Analysis of the correlation between the anti-angiogenic efficacy of combination therapy and the inhibition of HIF-1α. Data are presented as mean ± SD from three independent experiments. ns = not significant, **p* < 0.05, ****p* < 0.001.

**
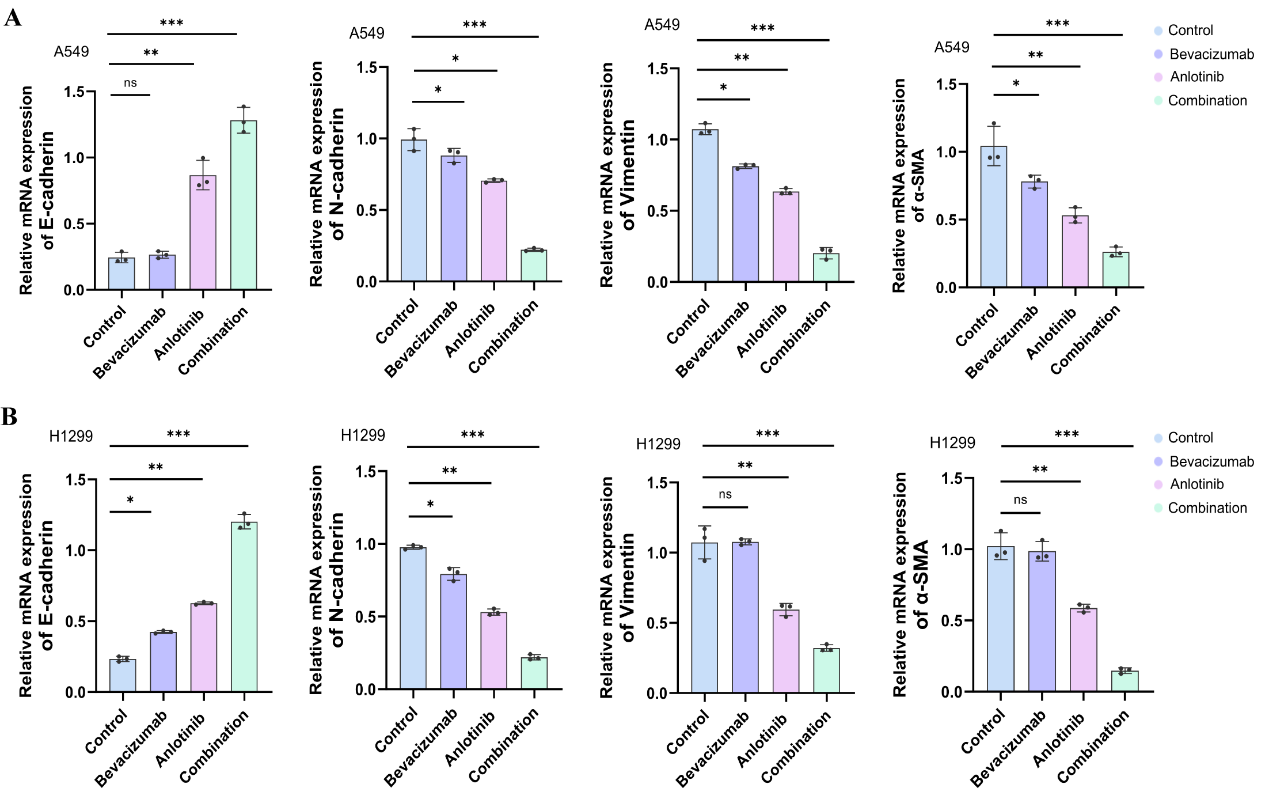
**

**Figure S6.** A. qRT-PCR measurements of EMT marker mRNA after 24 h treatment of A549 cells with anlotinib, bevacizumab or a combination of both; B. qRT-PCR measurements of EMT marker mRNA after 24 h treatment of H1299 cells with anlotinib, bevacizumab or a combination of both. Data are presented as mean ± SD from three independent experiments. ns = not significant, **p* < 0.05, ***p* < 0.01, ****p* < 0.001.


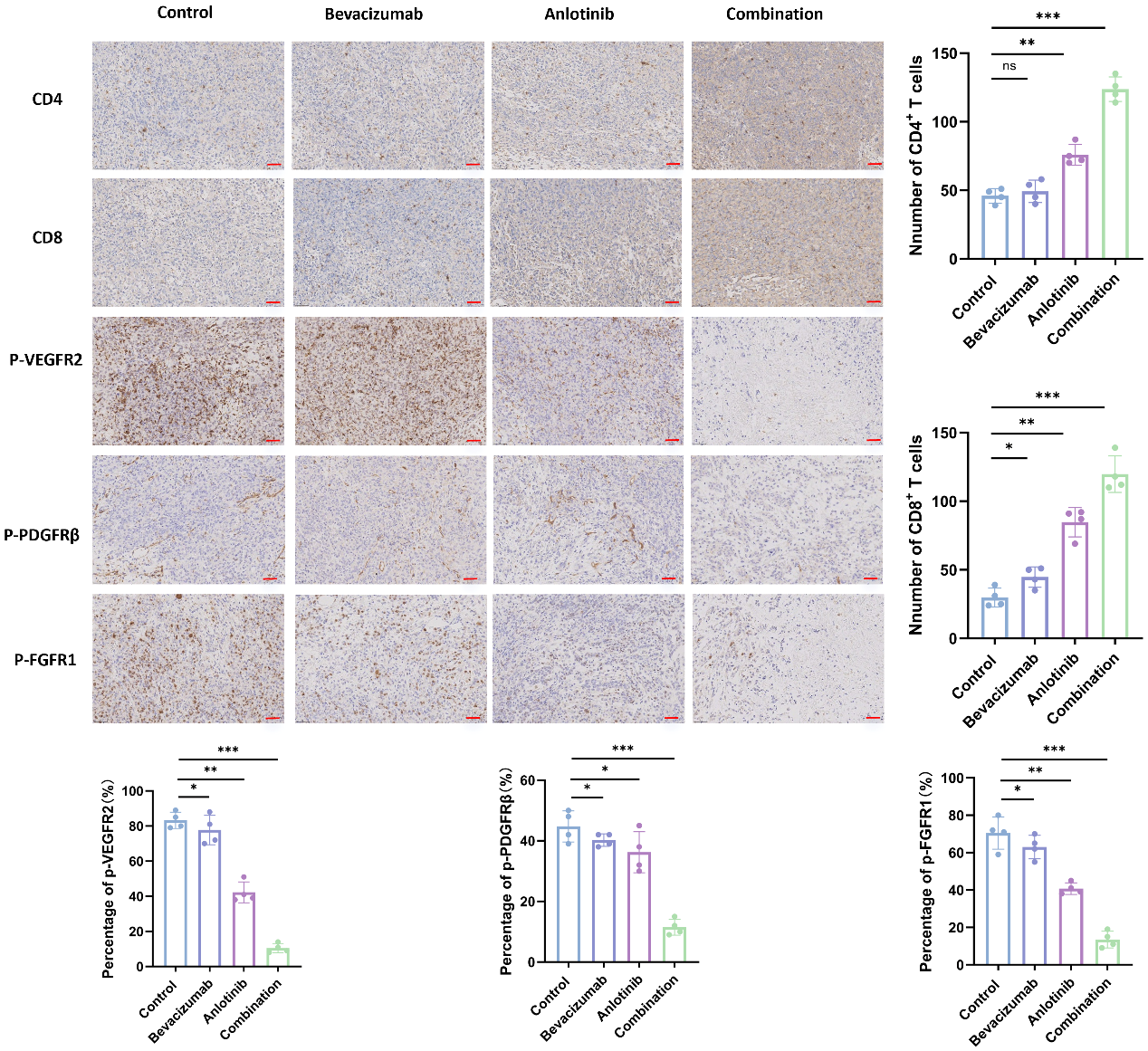


**Figure S7.** Immunohistochemical analysis was performed to assess immune cell infiltration and angiogenic receptor activation in tumor tissues from different treatment groups. Scale bar = 50μm. Data are presented as mean ± SD from three independent experiments. *p < 0.05, **p < 0.01, ***p < 0.001.


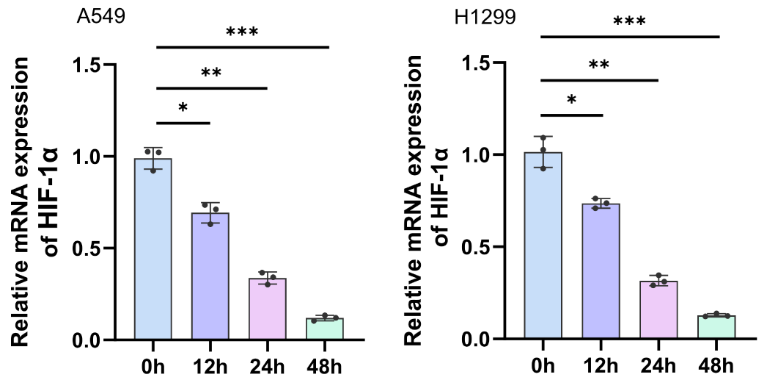


**Figure S8.** Quantitative RT-PCR analysis of HIF-1α mRNA levels in A549 and H1299 cells treated with the combination of anlotinib and bevacizumab for 0, 12, 24 and 48 h. Data are presented as mean ± SD from three independent experiments. **p* < 0.05, ***p* < 0.01, ****p* < 0.001.

**
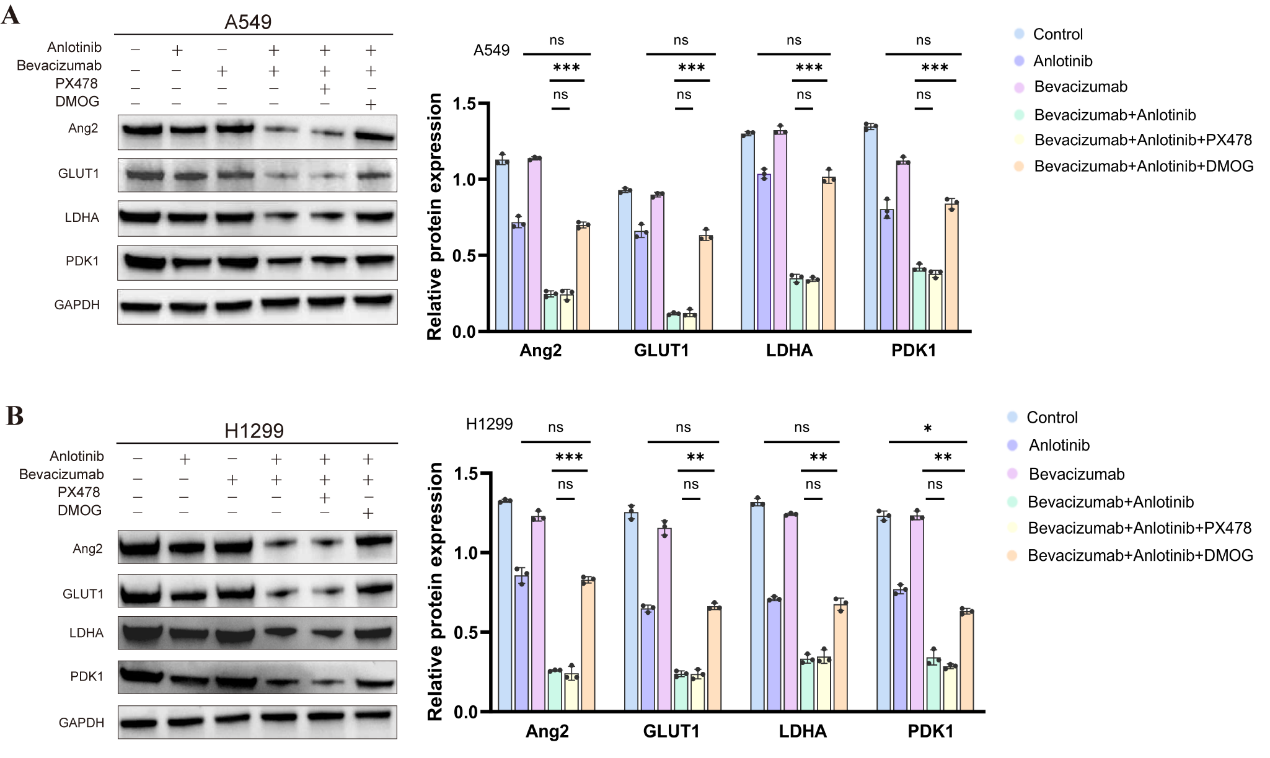
**

**Figure S9.** A. Western blotting of downstream HIF-1α targets, metabolic and angiogenic markers, Ang2, GLUT1, LDHA and PDK1, in A549 cells after 24 h of treatment; B. Western blotting of downstream HIF-1α targets, metabolic and angiogenic markers, Ang2, GLUT1, LDHA and PDK1, in H1299 cells after 24 h of treatment. Data are presented as mean ± SD from three independent experiments. ns = not significant, **p* < 0.05, ***p* < 0.01, ****p* < 0.001.


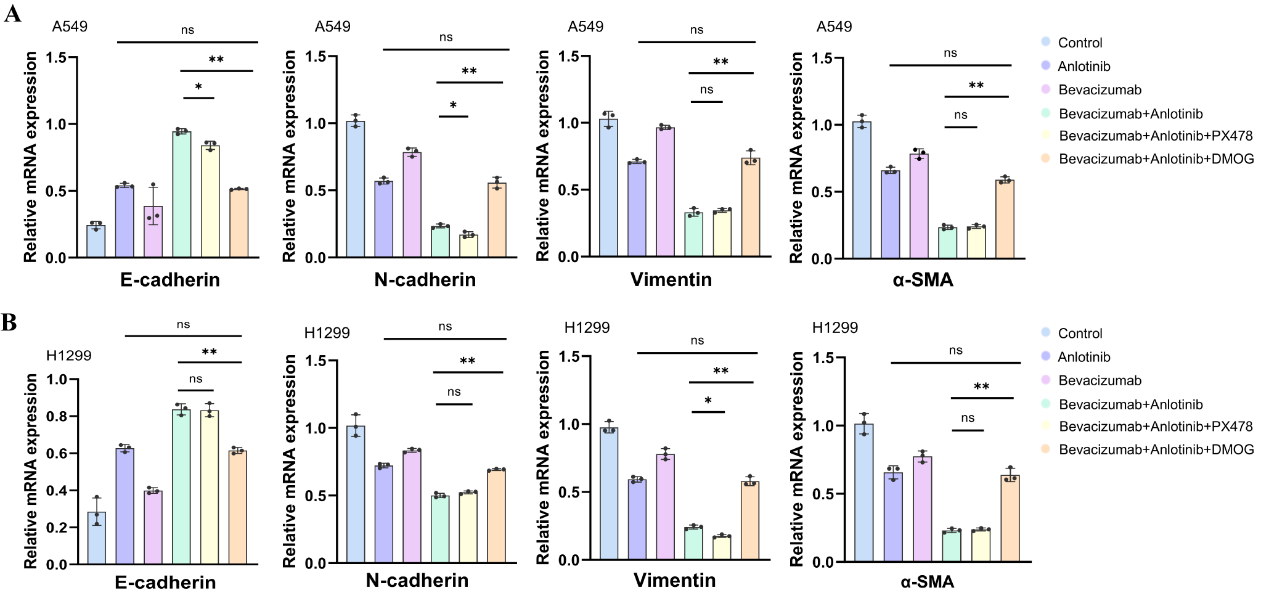


**Figure S10.** A. qRT-PCR measurements of EMT marker, E-cadherin, N-cadherin, vimentin and α-SMA, mRNA in A549 cells after 24 h of treatment; B. qRT-PCR measurements of EMT marker, E-cadherin, N-cadherin, vimentin and α-SMA, mRNA in H1299 cells after 24 h of treatment. Data are presented as mean ± SD from three independent experiments. ns = not significant, **p* < 0.05, ***p* < 0.01.


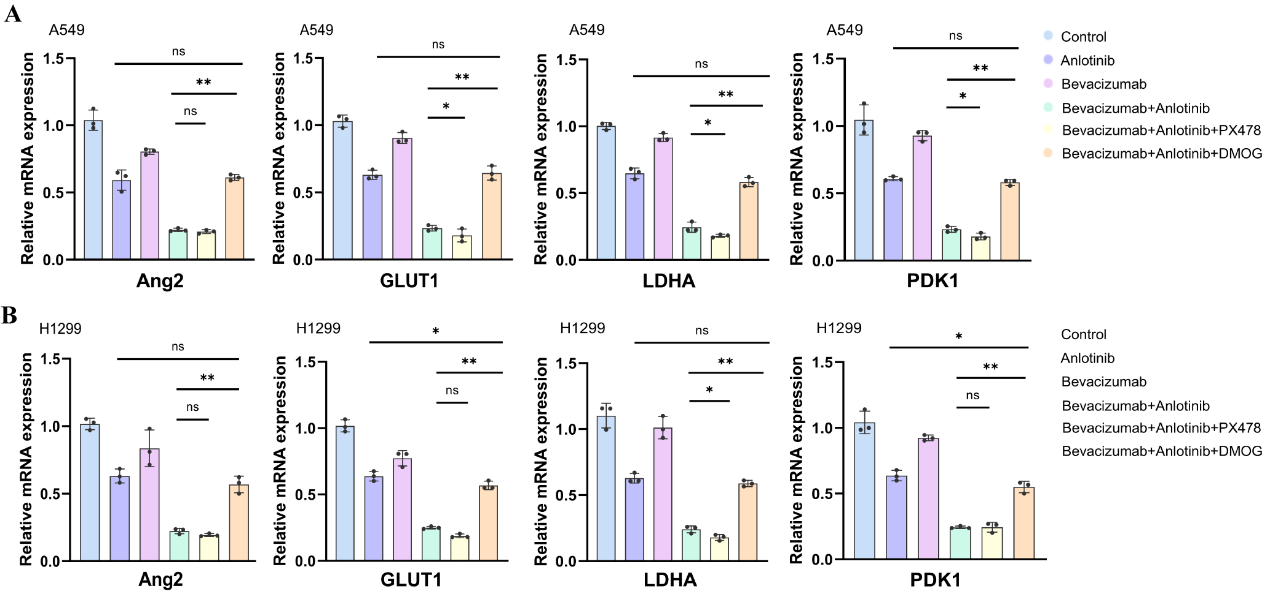


**Figure S11.** A. qRT-PCR measurement of mRNA of HIF-1α downstream metabolic targets, Ang2, GLUT1, LDHA and PDK1, in A549 cells after 24 h of treatment; B. qRT-PCR measurement of mRNA of HIF-1α downstream metabolic targets, Ang2, GLUT1, LDHA and PDK1, in H1299 cells after 24 h of treatment. Data are presented as mean ± SD from three independent experiments. ns = not significant, **p* < 0.05, ***p* < 0.01.
